# Reciprocal c-di-GMP signaling: Incomplete flagellum biogenesis triggers c-di-GMP signaling pathways that promote biofilm formation

**DOI:** 10.1371/journal.pgen.1008703

**Published:** 2020-03-16

**Authors:** Daniel C. Wu, David Zamorano-Sánchez, Fernando A. Pagliai, Jin Hwan Park, Kyle A. Floyd, Calvin K. Lee, Giordan Kitts, Christopher B. Rose, Eric M. Bilotta, Gerard C. L. Wong, Fitnat H. Yildiz

**Affiliations:** 1 Department of Microbiology and Environmental Toxicology, University of California, Santa Cruz, California, United States of America; 2 Department of Bioengineering, University of California, Los Angeles, California, United States of America; 3 Department of Chemistry and Biochemistry, University of California, Los Angeles, California, United States of America; 4 California Nano Systems Institute, University of California, Los Angeles, California, United States of America; Universidad de Sevilla, SPAIN

## Abstract

The assembly status of the *V*. *cholerae* flagellum regulates biofilm formation, suggesting that the bacterium senses a lack of movement to commit to a sessile lifestyle. Motility and biofilm formation are inversely regulated by the second messenger molecule cyclic dimeric guanosine monophosphate (c-di-GMP). Therefore, we sought to define the flagellum-associated c-di-GMP-mediated signaling pathways that regulate the transition from a motile to a sessile state. Here we report that elimination of the flagellum, via loss of the FlaA flagellin, results in a flagellum-dependent biofilm regulatory (FDBR) response, which elevates cellular c-di-GMP levels, increases biofilm gene expression, and enhances biofilm formation. The strength of the FDBR response is linked with status of the flagellar stator: it can be reversed by deletion of the T ring component MotX, and reduced by mutations altering either the Na^+^ binding ability of the stator or the Na^+^ motive force. Absence of the stator also results in reduction of mannose-sensitive hemagglutinin (MSHA) pilus levels on the cell surface, suggesting interconnectivity of signal transduction pathways involved in biofilm formation. Strains lacking flagellar rotor components similarly launched an FDBR response, however this was independent of the status of assembly of the flagellar stator. We found that the FDBR response requires at least three specific diguanylate cyclases that contribute to increased c-di-GMP levels, and propose that activation of biofilm formation during this response relies on c-di-GMP-dependent activation of positive regulators of biofilm production. Together our results dissect how flagellum assembly activates c-di-GMP signaling circuits, and how *V*. *cholerae* utilizes these signals to transition from a motile to a sessile state.

## Author summary

A key regulator of *Vibrio cholerae* physiology is the nucleotide-based, second messenger cyclic dimeric guanosine monophosphate (c-di-GMP). We found that the status of flagellar biosynthesis at different stages of flagellar assembly modulates c-di-GMP signaling in *V*. *cholerae* and identified diguanylate cyclases involved in this regulatory process. The effect of motility status on the cellular c-di-GMP level is partly dependent on the flagellar stator and Na^+^ flux through the flagellum. Finally, we showed that c-di-GMP-dependent positive regulators of biofilm formation are critical for the signaling cascade that connects motility status to biofilm formation. Our results show that in addition to c-di-GMP promoting motile to biofilm lifestyle switch, “motility status” of *V*. *cholerae* modulates c-di-GMP signaling and biofilm formation.

## Introduction

The ability of bacterial communities to form biofilms–multicellular aggregates encased by an extracellular matrix of polysaccharides, proteins, lipids and DNA–enhances environmental fitness and allows microorganisms to persist in different niches [1]. The initial stages of biofilm formation by flagellated bacteria require modulation of flagella-mediated motility [2]. The bacterial flagellum is built by a large set of proteins, and consists of a motor complex, which includes a rotor, a stator, and a rod, connected to the flagellum filament by a hook structure [3–5]. The rotation of the flagellum is powered by an ion motive force that fuels the flagellum-motor complex. Flagellar function during biofilm formation can be regulated at two stages: the assembly stage, via modulation of a series of transcriptional regulators; and at the post-assembly stage, via interactions between effector proteins and motor proteins [2].

A key regulator of the transition between a motile state and a biofilm state is the second messenger cyclic-dimeric guanosine monophosphate (c-di-GMP) [6,7]. Production of c-di-GMP is controlled by diguanylate cyclases (DGCs) and phosphodiesterases (PDEs) [8–11]. High c-di-GMP levels inhibit motility, and studies have elucidated some of the mechanisms involved [6]. These include repressing transcription of flagellar genes or acting post-transcriptionally to regulate flagellar reversals and/or speed either by interacting with specific flagellar-motor proteins or by altering the chemotactic signal-transduction system [12–17].

**Fig 1:**
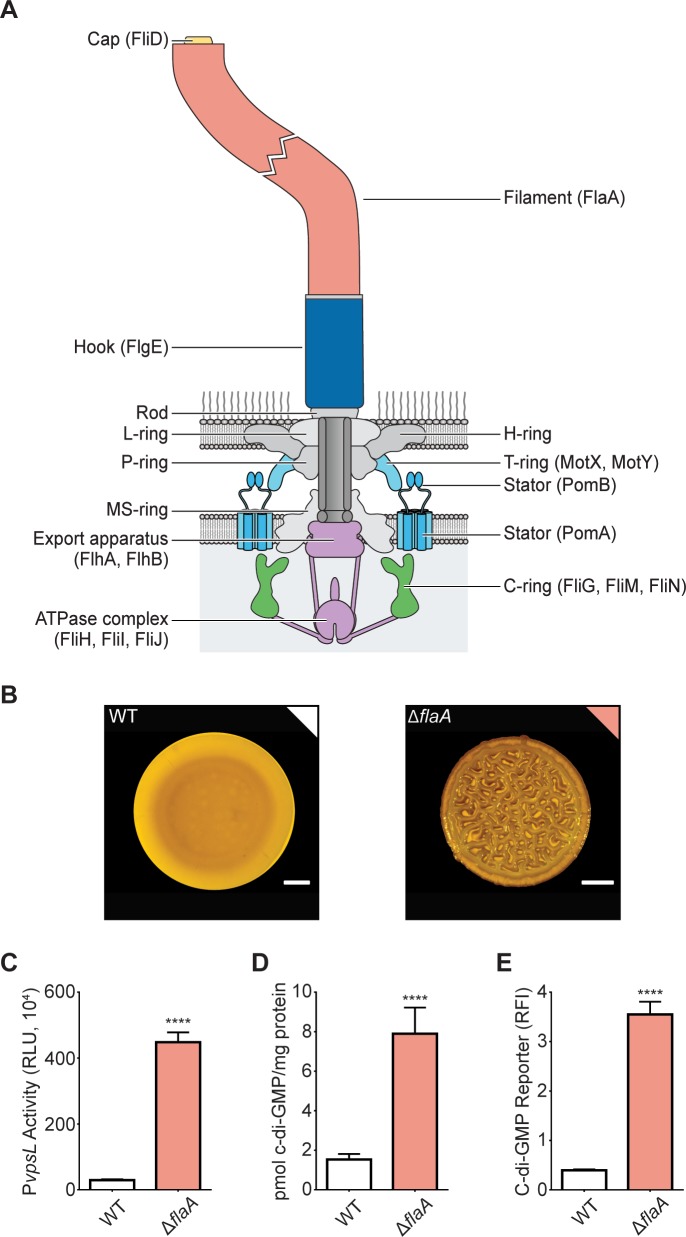
The absence of the *V*. *cholerae* flagellum filament elicits a flagellum-dependent biofilm regulatory response. A) Illustration showing the main components of the polar flagellum in *V*. *cholerae* with the proteins forming these components shown in brackets. The flagellum sheath is not depicted in this figure. The structures targeted in this study are color coded. B) Representative images of the smooth colony morphology of the WT strain and the corrugated colony morphology of the Δ*flaA* strain. Scale bars = 1 mm. C) Bar graph of means and standard deviations of relative luminescent units (RLU) obtained from the transcription of *vpsL*-*luxCDABE* in colonies of the WT and Δ*flaA* strains. D) Bar graph of means and standard deviations of c-di-GMP concentration measured by LC-MS/MS in colonies of the WT and Δ*flaA* strains. E) Bar graph of means and standard deviations of RLU obtained from the expression of the c-di-GMP biosensor in colonies of the WT and Δ*flaA* strains. Means obtained from three biological replicates were compared with an unpaired t-test. Mean differences with a P value ≤ 0.05 were deemed significant. **** p ≤ 0.0001.

*Vibrio cholerae*, the causal agent of the diarrheal disease cholera, is motile via the action of a single polar-sheathed flagellum that is powered by the Na^+^ motive force [18]. The *Vibrio* flagellum contains a stator comprised of PomA and PomB, along with periplasmic H and T rings (Fig 1A) that are not present in flagella powered by H^+^ motive forces [19–21]. The flagellar T ring (composed of MotX and MotY) is required for torque generation and recruitment of the stator components, and interacts directly with the stator component PomB [22]. The biogenesis of the *V*. *cholerae* flagellum is regulated by a four-tiered transcriptional hierarchy that enables stepwise production of the building blocks required for an ordered flagellum assembly process [19,23–25]. c-di-GMP regulates flagellar motility both transcriptionally, by allosteric inhibition of the master flagellar transcriptional regulator FlrA, and post-translationally, via the c-di-GMP receptor MshE controlling the abundance of type IVa mannose-sensitive hemagglutinin (MSHA) pili on the cell surface and regulating the transition from motile to biofilm lifestyle in part by impacting flagellum-mediated near-surface motility [16, 26–28]. *V*. *cholerae* has four conserved PilZ-domain proteins, which regulate flagellar motility in other bacteria by interacting with flagellum motor components [12–14]. When one or more of these proteins are absent, there are modest defects in motility through mechanisms that are not yet understood [29,30].

*V*. *cholerae* biofilm formation requires production of biofilm-matrix components, polysaccharides and proteins that connect cells to each other and to biotic and abiotic surfaces [31–34]. The *Vibrio* polysaccharide (VPS) is required for biofilm formation and is synthesized from *vps* genes that are clustered in two regions on the large chromosome of *V*. *cholerae* O1 [35,36]. Additionally, three matrix proteins, RbmA, RbmC, and Bap1 are needed to form mature biofilms [37,38]. Enhanced production of biofilm matrix components VPS and matrix proteins results in formation of corrugated colonies. Transcription of biofilm genes is activated by VpsR and VpsT [39,40], which can both bind c-di-GMP; however, only VpsT requires c-di-GMP for its activity [41,42]. Expression of biofilm matrix genes can be repressed by the master quorum-sensing regulator HapR, along with the histone-like nucleoid structuring protein (H-NS) [43–49].

A connection between flagellum biogenesis and function and biofilm formation was previously demonstrated in *V*. *cholerae*. The absence of the major flagellin FlaA renders *V*. *cholerae* cells non-flagellated and non-motile, and promotes *vps* gene expression, which in turn enhances biofilm matrix production and formation of corrugated colonies [50,51]. This response is dependent on the presence of the stator [51], suggesting that *V*. *cholerae* cells have signaling circuits that connect both the presence and the activity of the flagellum to biofilm formation.

In this study, we first demonstrate that the regulation of biofilm formation by flagellum filament assembly involves changes in cellular c-di-GMP accumulation. The phenotypes observed in strains lacking the flagellar filament required a functional stator and to a lesser degree a functional Na^+^ translocating NADH:quinone oxidoreductase. Both the flagellar filament and the flagellar stator play important roles in regulating surface colonization and c-di-GMP accumulation during the initial stages of biofilm formation. The absence of the filament as well as the absence of flagellar rotor and export machinery components resulted in increased c-di-GMP accumulation and the formation of corrugated colonies. The phenotypes associated with the lack of flagellum basal body components was not affected by the absence of the flagellar stator. We additionally identified three DGCs governing c-di-GMP signaling involved in responding to incomplete flagellum biogenesis. Finally, we found that activation of the VpsR-VpsT regulatory cascade plays a more direct role than inactivation of the biofilm repressor HapR in promoting biofilm formation and c-di-GMP accumulation associated with incomplete flagellum biogenesis. Together our analyses reveal key elements of a multifaceted regulatory system that allows *V*. *cholerae* to connect different stages of flagellum biosynthesis with biofilm development using c-di-GMP as an intermediary.

## Results

### Absence of the major flagellin FlaA promotes c-di-GMP accumulation in *V*. *cholerae*

A *V*. *cholerae* Δ*flaA* strain lacking the main filament subunit forms colonies with a corrugated morphology compared to the smooth colonies of the wild-type (WT) strain (Fig 1B), and exhibits increased expression of the VPS biosynthetic operon II (*vpsL-Q*, pBBR-P*vpsL*-*lux*) (Fig 1C), although the molecular mechanisms involved have not been fully elucidated [50,51]. The expression of the *vps* genes is positively regulated by the second messenger c-di-GMP [52–54]. Thus, to gain insight into the mechanisms by which the lack of flagellum enhances biofilm formation, we measured cellular c-di-GMP levels in WT and Δ*flaA* biofilms. c-di-GMP abundance was 5-fold higher when analyzed using LC-MS/MS, and 9-fold higher when analyzed using a c-di-GMP genetic reporter in the Δ*flaA* strain compared to the WT strain (Fig 1D and 1E). These findings suggest that flagellum assembly triggers a flagellum-dependent biofilm regulatory response, hereafter referred as the FDBR response, which is characterized by an increase in biofilm gene expression, c-di-GMP production, and colony corrugation.

### The stator modulates cellular c-di-GMP levels and is required for the FDBR response in the Δ*flaA* strain

**Fig 2:**
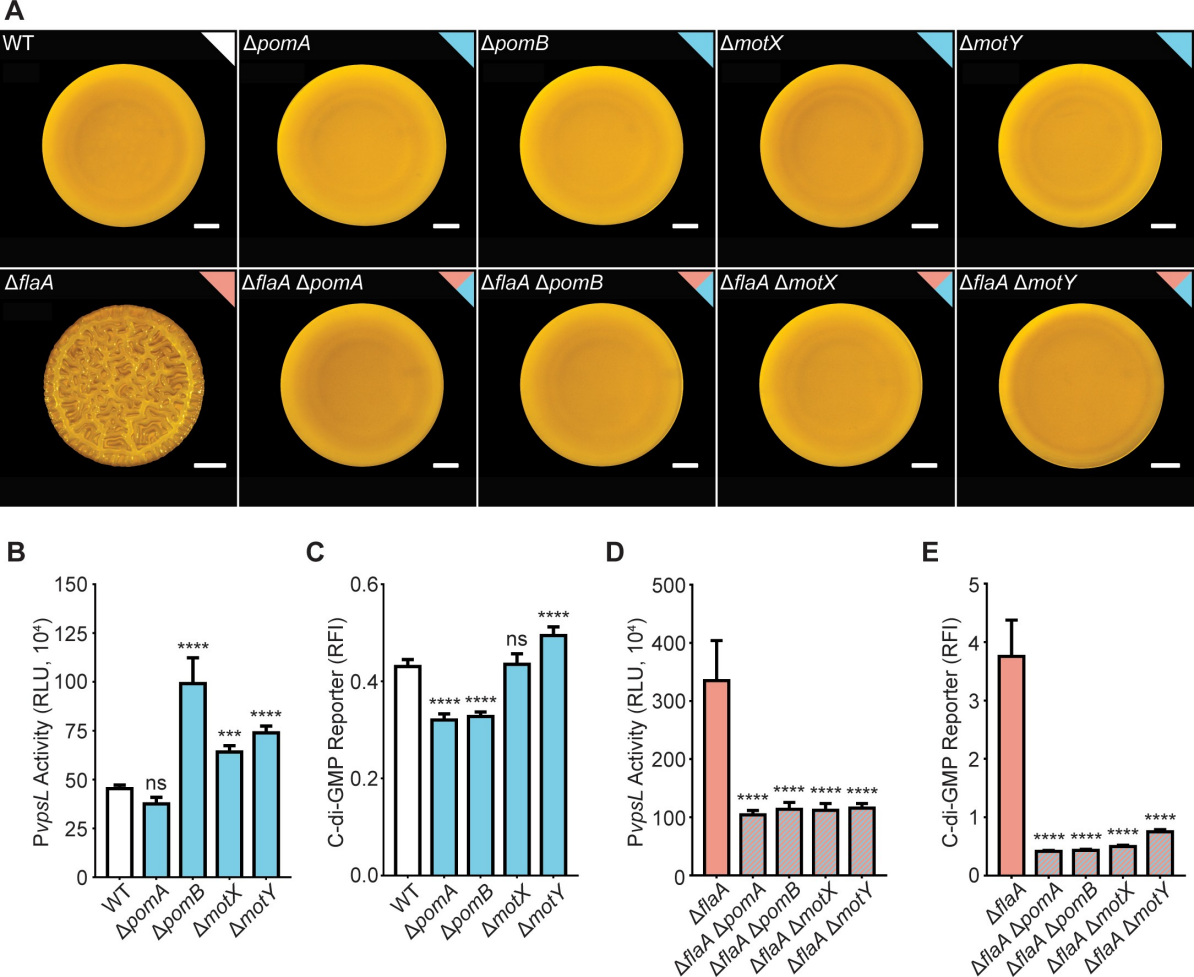
The FDBR response in the Δ*flaA* strain requires the presence of the stator and T ring. A) Representative images of the colony morphologies of the WT strain and strains lacking genes encoding stator and T-ring components in a WT or Δ*flaA* genetic background. Scale bars = 1 mm. B) Bar graph of means and standard deviations of RLU obtained from the transcription of *vpsL*-*luxCDABE* in colonies of the WT and single mutants lacking stator and T-ring genes. C) Bar graph of means and standard deviations of relative fluorescence intensity (RFI) obtained from the expression of the c-di-GMP biosensor in the WT and single mutants lacking stator and T-ring genes. D) Bar graph of means and standard deviations of RLU obtained from the transcription of *vpsL*-*luxCDABE* in colonies of the Δ*flaA* strain and Δ*flaA* double mutants lacking stator and T-ring genes. E) Bar graph of means and standard deviations of RFI obtained from the expression of the c-di-GMP biosensor in colonies of the Δ*flaA* strain and Δ*flaA* double mutants lacking stator and T-ring genes. Means obtained from 3 biological replicates were compared to WT or Δ*flaA* with a one-way ANOVA followed by Dunnett’s multiple-comparison test. Adjusted P values ≤ 0.05 were deemed significant. *** p ≤ 0.001; **** p ≤ 0.0001. ns not significant. The color of each bar represents the type of flagellum structure to which each gene product belongs as depicted in Fig 1A.

The increased *vps* expression and colony corrugation exhibited by strains lacking the flagellum filament requires the presence of a functional stator [50,51]. This is notable because the stator has been proposed to serve as a mechano-sensor in multiple bacterial species [55–57], hence mechano-sensation could be associated with c-di-GMP signaling in *V*. *cholerae*. To evaluate the contribution of the stator components (PomA and PomB) and the T ring components to the FDBR response, we generated in-frame deletions in *pomA* and *pomB*, which encode the Na^+^-driven motor, and in *motX* and *motY*, which are T ring components, in WT and Δ*flaA* genetic backgrounds. In the WT background, there was no difference in colony morphology between the single mutants and the WT strain (Fig 2A), and only modest changes in expression from the *vps*-II operon and in c-di-GMP levels (Fig 2B and 2C). In contrast, in the Δ*flaA* background, the colony morphologies of the double-mutant strains (Δ*flaA*Δ*pomA*, Δ*flaA*Δ*pomB*, Δ*flaA*Δ*motY*, and Δ*flaA*Δ*motX*) were smooth as opposed to the corrugated colony morphology of the Δ*flaA* strain (Fig 2A), and the loss of corrugation was accompanied by significantly decreased expression from the *vps*-II operon and by decreased c-di-GMP accumulation (Fig 2D and 2E). Thus, the FDBR response, triggered by the absence of *flaA*, depends on the presence of the flagellum stator and its assembly.

### Point mutations in PomB that alter Na^+^ binding and deletion of *nqrB* and *nqrC* suppress the FDBR response in the Δ*flaA* strain

**Fig 3:**
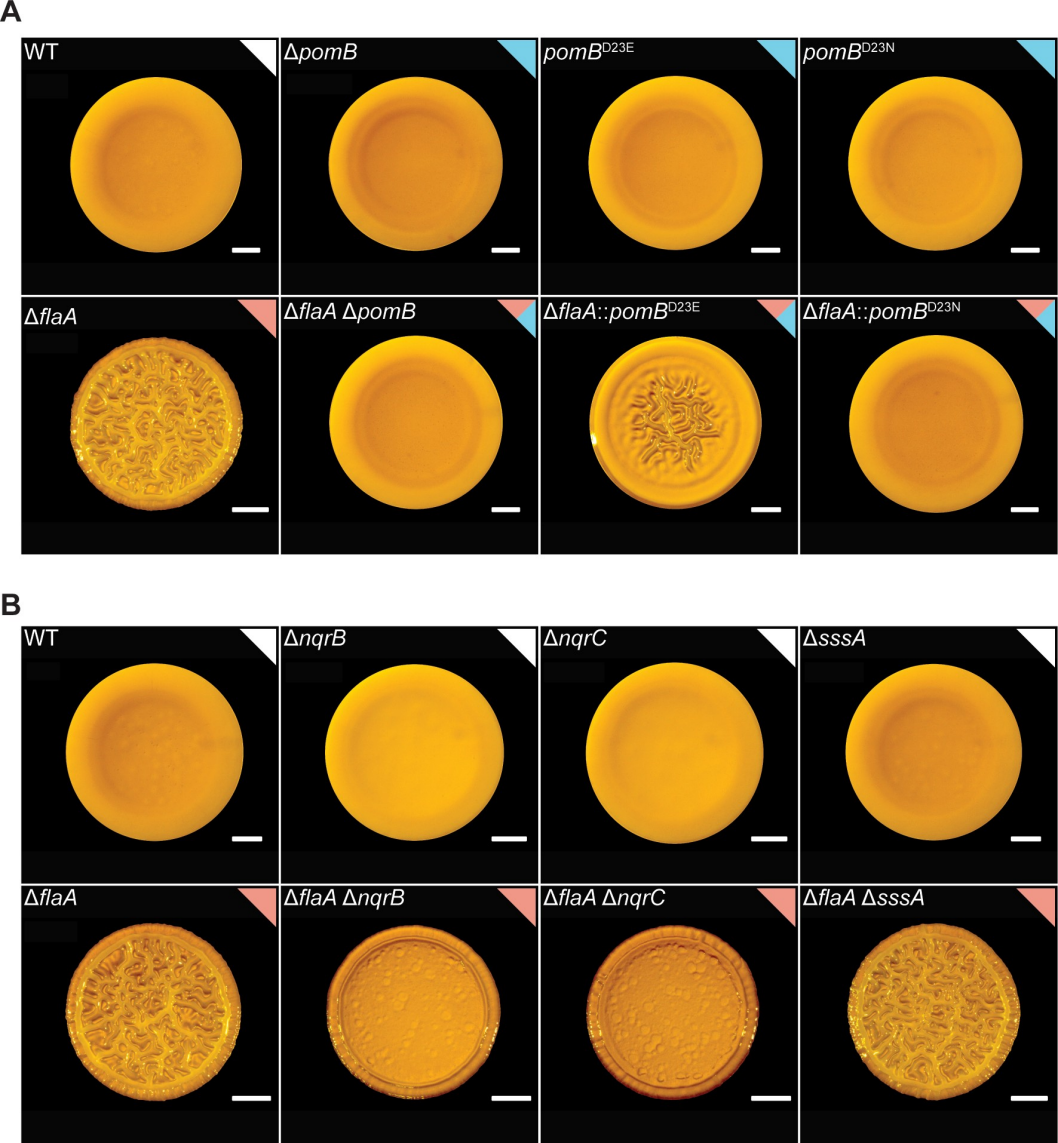
In the Δ*flaA* strain, PomB variants with defects in Na^+^ binding or absence of Na^+^-NQR components impact colony corrugation. A) Representative images of the colony morphologies of strains with mutations in *pomB* in WT and Δ*flaA* backgrounds. B) Representative images of the colony morphologies of strains lacking subunits of the Na^+^-NQR complex or the Na^+^ symporter SssA in the WT and Δ*flaA* backgrounds. Experiments were performed on 3 biological replicates. Scale bars = 1 mm.

To gain insight into the mechanisms by which the stator participates in the FDBR response, we mutated PomB at the conserved aspartate residue at position 23, which is predicted to affect its affinity for Na^+^ and thereby stator function [58]. Colony morphologies of the PomB^D23E^ or PomB^D23N^ strains were indistinguishable from the WT strain (Fig 3A). In contrast, in the Δ*flaA* strain, PomB^D23N^ eliminated colony corrugation, while PomB^D23E^ reduced it (Fig 3A). These results suggest that an impairment in Na^+^ transport by PomB negatively affects the FDBR response in the Δ*flaA* strain.

The *V*. *cholerae* flagellum is powered by Na+ ions. The NQR complex is required to maintain the sodium motive force, which has been shown to impact flagellum stator assembly in the related bacterium *Vibrio alginolyticus* [59,60]. Thus, we evaluated whether the NQR complex regulates the FDBR response. To test this, we generated mutations in subunits of the Na^+^-translocating NADH:quinone oxidoreductase Na^+^-NQR in both a WT and a Δ*flaA* genetic background. The single deletion of *nqrB* or *nqrC* did not affect colony corrugation when compared to the WT strain (Fig 3B). In contrast, the Δ*flaA*Δ*nqrB* and Δ*flaA*Δ*nqrC* strains had markedly reduced colony corrugation compared to the Δ*flaA* strain and formed more compact colonies compared to the WT strain (Fig 3B). Since Na^+^-NQR is important for the ion motive force and membrane potential [59], we propose that the electric state of the membrane is important for the FDBR response. It is notable that the absence of either the NqrB or NqrC subunits of the Na^+^-NQR pump was less detrimental to colony corrugation than the absence of the flagellar stator.

We additionally evaluated the impact of the lack of *sssA*, which encodes a sodium symporter, in both the WT and Δ*flaA* strains. The SssA pump, like the Na^+^-NQR pump, is involved in the transition from transient to permanent attachment in *V*. *cholerae* biofilms [61]. The Δ*sssA* and Δ*flaA* Δ*sssA* strains showed colony corrugation indistinguishable from their respective WT and Δ*flaA* genetic backgrounds (Fig 3B). This suggests that this Na^+^ symporter is not required for the FDBR response triggered in the Δ*flaA* strain.

### The absence of the flagellum filament and/or the flagellum stator alters dynamics of biofilm formation and c-di-GMP accumulation

**Fig 4:**
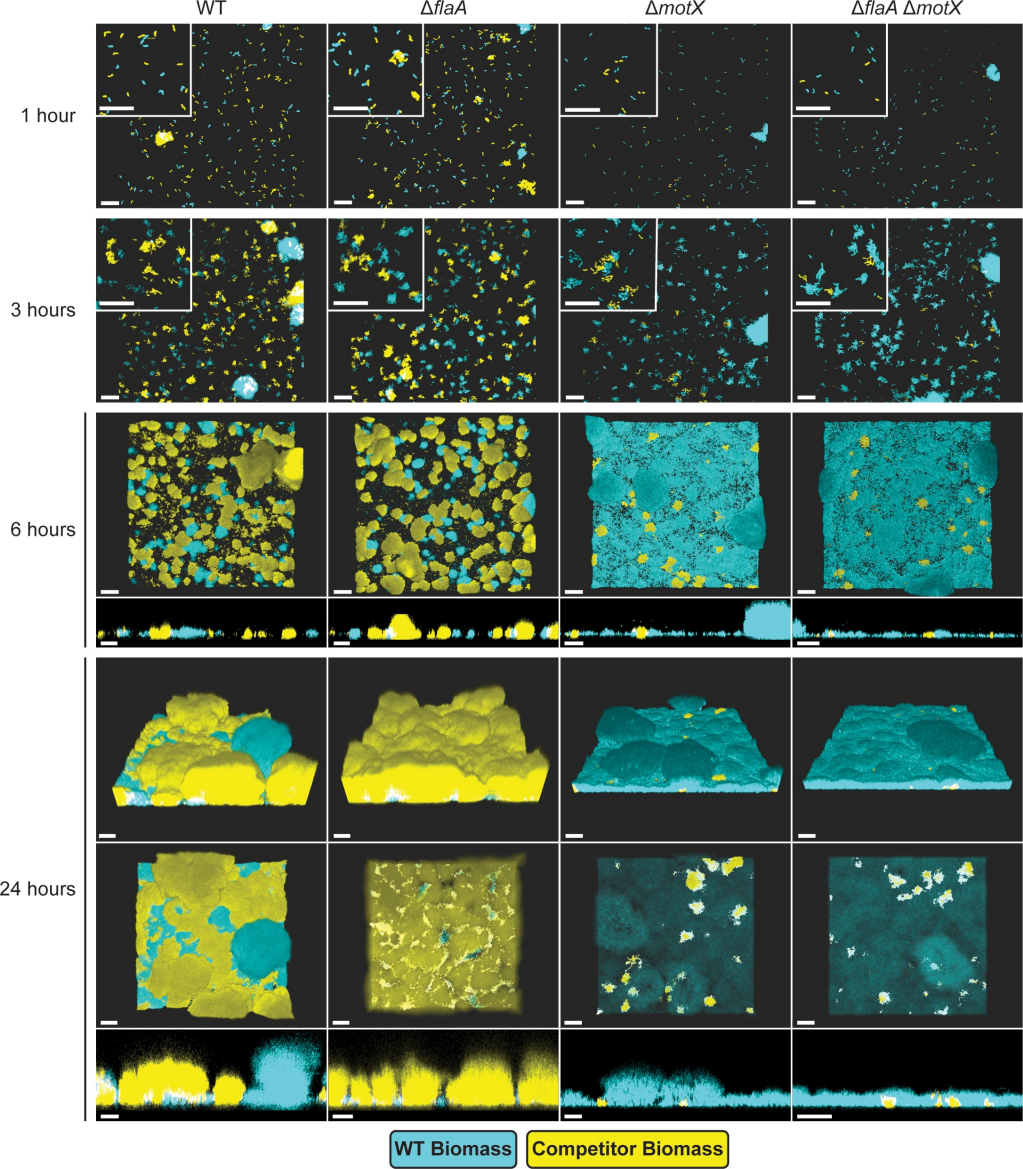
The absence of the flagellum filament and/or stator influences biofilm formation. Representative images of flow cell biofilm competition experiments using 1:1 mixtures of WT-RFP (cyan) and WT-GFP or mutant-GFP (yellow) strains. Images were obtained at 40x magnification at stages typical of initial surface attachment (1 hour, 1H), microcolony development (6 hours, 6H), and mature biofilm (24 hours, 24H). Images were generated using Imaris software. Insets in the upper left corners of 1- and 3-hour images are magnifications of regions from the same image that depict single cells and initial microcolonies. Cross sections of the XZ planes are shown for images taken at 6 and 24 hours. Images are representative of a minimum of 3 biological replicates per strain with three technical replicate images obtained per biological replicate at each time point. Scale bars = 20 μm.

We have shown that the Δ*flaA*, Δ*motX*, and Δ*flaA*Δ*motX* strains have altered FDBR responses. This prompted us to investigate the abilities of these strains to compete with the WT strain for biofilm formation under constant flow in a microfluidic chamber. We utilized a WT strain fluorescently tagged with RFP, and Δ*flaA*, Δ*motX*, and Δ*flaA*Δ*motX* strains tagged with GFP. We analyzed biofilm formation at time-points representing the stages of monolayer formation (1 hour), initial and mature microcolony formation (3 and 6 hours, respectively), and mature biofilm (24 hours), using a 1:1 ratio of WT to mutant strain. Biofilm formation was quantified using the software COMSTAT2; data are summarized in S1 Table. As a control we evaluated biofilm formation by a 1:1 mixture of WT-GFP and WT-RFP and observed similar surface colonization and biofilm formation properties for the two strains (Fig 4).

In the competition assay, the Δ*flaA* strain showed no defects in surface attachment and monolayer formation compared to WT, and formation of microcolonies was not significantly different between the Δ*flaA* and WT strains after 3 hours (Fig 4). However, at 6 hours, the Δ*flaA* strain showed enhanced development of mature microcolonies compared to WT. This enhancement persisted through the later stages of biofilm formation, and by 24 hours the Δ*flaA* strain had outcompeted the WT strain (Fig 4).

Although loss of the flagellar filament caused no defect in surface attachment, loss of the stator in Δ*motX* and Δ*flaA*Δ*motX* strains considerably impaired surface attachment and monolayer formation compared to WT (Fig 4). Cells of the Δ*motX* and Δ*flaA*Δ*motX* strains that did attach to the surface were able to form microcolonies and small biofilm structures (Fig 4). However, by 24 hours the majority of the biomass in these biofilms corresponded to the WT strain. We conclude that the Δ*motX* and Δ*flaA*Δ*motX* strains are defective in surface attachment and microcolony formation, which impairs their downstream ability to form mature biofilms. Biofilms formed by mixed populations of the WT and the Δ*motX* or Δ*flaA*Δ*motX* strains had less biofilm biomass and thickness compared to the mixed populations of the WT-GFP vs. WT-RFP control and WT vs. Δ*flaA* strains (Fig 4). Together, these results show that the Δ*flaA* strain is capable of outcompeting the WT strain without alteration of surface attachment, whereas the Δ*motX* and Δ*flaA*Δ*motX* strains are readily outcompeted by the WT strain due to defects in surface attachment.

**Fig 5:**
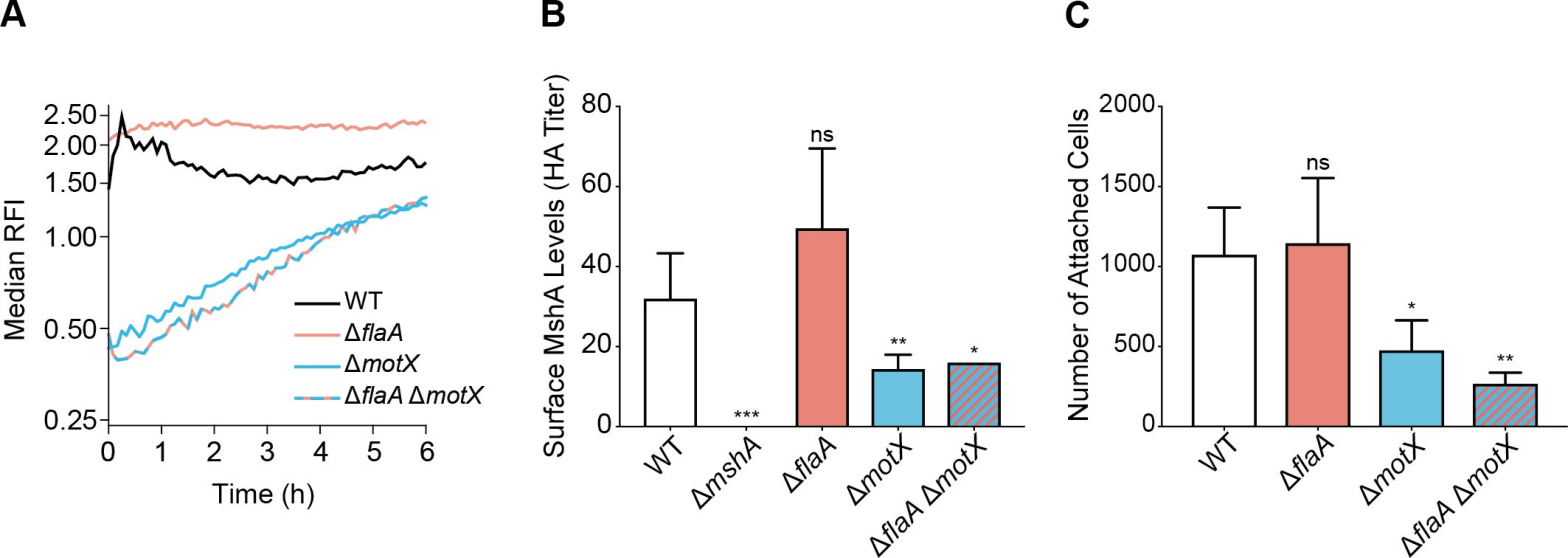
The absence of the flagellum filament and/or stator alters the dynamics of c-di-GMP accumulation and MSHA-surface abundance. A) Plot of the median RFI for individual WT, Δ*flaA*, Δ*motX*, and Δ*flaA* Δ*motX* cells attached to the surface inside flow cells. For each time point and strain, the distribution of RFI values were obtained from 2 independent experiments, and the median was calculated from these distributions. Time t = 0 h corresponds to the start of image acquisition after flow started, not inoculation time, for a more unbiased comparison of surface attached cells between strains with and without attachment defects. Error estimates for these RFI values, in the form of 95% confidence intervals, are shown in supplementary S1 Fig. B) Surface MshA levels determined by MSHA-specific hemagglutination (HA) assay. The HA titer is defined as the reciprocal of the lowest dilution at which agglutination of sheep erythrocytes was observed for each strain. Equivalent cell numbers were used for each strain, normalized by OD_600_. Bar graph of means with standard error of the mean of MSHA-specific HA titer. Data were obtained from 5 biological replicates, with 2 technical replicates for each biological replicate per strain. Each mutant HA titer compared to WT via unpaired two-tailed Student’s *t*-Test, Δ*mshA* ***p = 0.0002, Δ*flaA* ns *p* = 0.1241, Δ*motX* **p = 0.0106, Δ*flaA*Δ*motX* *p = 0.0133. C) Analysis of surface-attachment ability. A total of 4 biological replicates were analyzed for each strain, and data is presented as mean with the standard deviation. Each mutant compared to WT via unpaired two-tailed Student’s *t*-Test, Δ*flaA* ns *p* = 0.7842, Δ*motX* *p = 0.0141, Δ*flaA*Δ*motX* **p = 0.0018.

We next analyzed whether the absence of the filament and/or the stator affect c-di-GMP accumulation at early stages of biofilm formation in flow cells. To test this, we used a stably expressed fluorescent c-di-GMP reporter and analyzed c-di-GMP accumulation dynamics over 6 hours in single cells attached to flow cell chambers in WT, Δ*flaA*, Δ*motX*, and Δ*flaA*Δ*motX* strains. In the WT strain, there was a rapid increase in c-di-GMP during the first 30 minutes followed by a return to basal levels (Fig 5A and S1 Fig). In contrast, cells from the Δ*flaA* strain showed higher basal levels of c-di-GMP compared to the WT strain that remained relatively constant over the 6 hours. In the Δ*motX* and Δ*flaA*Δ*motX* strains, basal c-di-GMP levels were approximately 3-fold lower than the WT, and although they gradually accumulated over 6-hours, levels remained below those in the WT strain (Fig 5A and S1 Fig). These observations further indicate that the flagellum filament and the flagellum stator play opposite roles in controlling c-di-GMP dynamics in surface-attached cells during initial stages of biofilm formation, and that the absence of the stator is dominant over the absence of the filament with respect to these phenotypes.

### The absence of the flagellum stator reduces MSHA pilus levels on the cell surface

In *V*. *cholerae* O1 El Tor strains, production of the type IV MSHA pilus is essential for the colonization of abiotic surfaces. We speculated that production of the MSHA pilus might also be regulated in response to the state of flagellum assembly. To test this, we analyzed the levels of MSHA pili on the cell surface using a hemagglutination (HA) assay with sheep erythrocytes. HA of sheep erythrocytes is specific to the MSHA pilus as it was blocked by deletion of the major pilin subunit (Δ*mshA*) (Fig 5B). There was no significant difference in HA ability between WT and Δ*flaA* strains (Fig 5B). However, loss of the flagellum stator in the Δ*motX* and Δ*flaA*Δ*motX* strains significantly reduced HA ability compared to WT (Fig 5B). Cell surface MSHA levels were analyzed from cells grown to mid-exponential phase, where we have previously observed MSHA production to be at its peak [27]. Analysis of the ability of each strain to attach to a surface under the same conditions yielded results correlative with the HA titers, where only Δ*motX* and Δ*flaA*Δ*motX* strains showed significant defects in attachment compared to WT (Fig 5C). Collectively, these findings indicate that reduction in cell surface MSHA production within the Δ*motX* and Δ*flaA*Δ*motX* strains, mediates the corresponding decrease in surface attachment and down-stream biofilm fitness (Fig 4).

### Three DGCs are necessary to trigger the FDBR response in the Δ*flaA* strain

**Fig 6:**
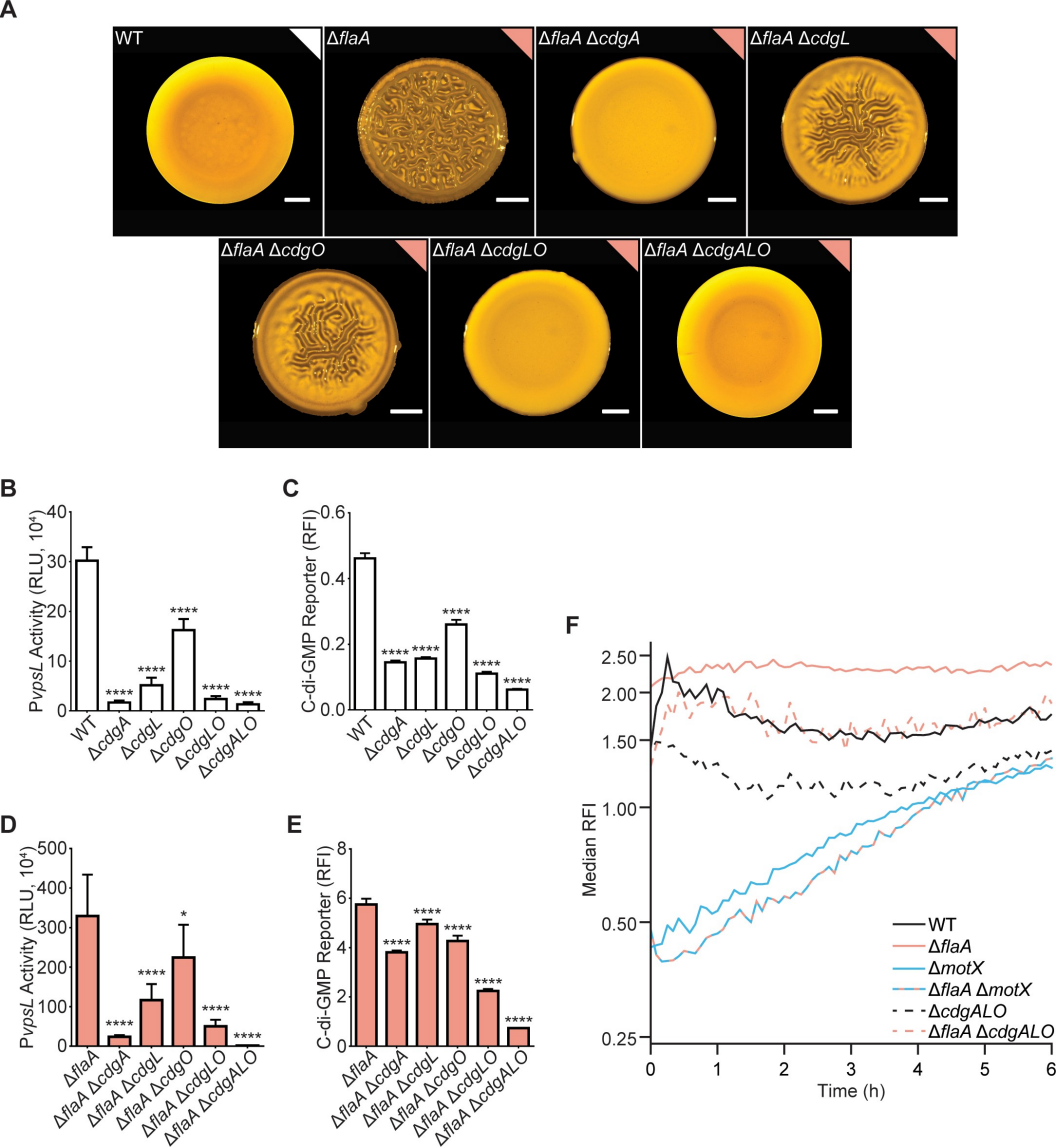
Three DGCs are required for the FDBR response in the Δ*flaA* strain. A) Representative images of the colony morphologies of indicated strains. Scale bars = 1 mm. B) Bar graph of means and standard deviations of RLU obtained from the transcription of *vpsL*-*luxCDABE* in colonies of indicated strains. C) Bar graph of means and standard deviations of RFI obtained from the expression of the c-di-GMP biosensor in indicated strains. D) Bar graph of means and standard deviations of RLU obtained from the transcription of *vpsL*-*luxCADBE* in colonies of indicated strains. E) Bar graph of means and standard deviations of RFI obtained from the expression of the c-di-GMP biosensor in indicated strains. F) Plots of the median RFI for Δ*cdgALO* and Δ*flaA* Δ*cdgALO* cells on the surface in flow cells. Data from the WT, Δ*flaA*, Δ*motX*, and Δ*flaA* Δ*motX* strains are the same as in Fig 5. Flow cell experiments from Fig 5 and Fig 6 were done in parallel and separated for clarity. For each time point and strain, the distribution of RFI values were obtained from 2 independent experiments, and the median was calculated from these distributions. Time t = 0 h corresponds to when image acquisition began after flow started, not inoculation time for a more unbiased comparison of surface attached cells between strains with and without attachment defects. Error estimates for these RFI values, in the form of 95% confidence intervals, are shown in supplementary S1 Fig. Means were compared to WT or Δ*flaA* with a one-way ANOVA followed by Dunnett’s multiple-comparison test. Adjusted P values ≤ 0.05 were deemed significant. * p ≤ 0.05; **** p ≤ 0.0001. Experiments were done on 3 biological replicates.

As the Δ*flaA* strain accumulates more c-di-GMP than the WT strain (Fig 1), we hypothesized that increased c-di-GMP could be dependent on one of the 28 DGCs with a conserved GGDEF domain. We evaluated the contribution of each of the 28 DGCs by deleting their corresponding genes in the Δ*flaA* genetic background and analyzing colony corrugation phenotypes. This revealed three DGCs required for colony corrugation in the Δ*flaA* strain (S2 Fig). The Δ*flaA*Δ*cdgA* strain formed more compact colonies than the WT strain but completely lacked colony corrugation (Fig 6A). The Δ*flaA*Δ*cdgL* and Δ*flaA*Δ*cdgO* strains formed colonies with markedly less corrugation compared to those formed by the Δ*flaA* strain (Fig 6A). The Δ*flaA*Δ*cdgL*Δ*cdgO* (Δ*flaA*Δ*cdgLO*) colonies resembled those from the Δ*flaA*Δ*cdgA* strain, whereas the Δ*flaA*Δ*cdgA*Δ*cdgL*Δ*cdgO* (Δ*flaA*Δ*cdgALO*) strain formed colonies indistinguishable from WT colonies (Fig 6A).

CdgA and CdgL are required for the expression of *vps* genes [45,62]; however, the involvement of CdgO in *vps* gene expression has not been established. We analyzed the effects of the lack of *cdgA*, *cdgL*, and *cdgO* both individually and in different combinations on the expression of the *vps*-II operon (pBBR-P*vpsL*-*lux*). The expression of *vps*-II was higher in the WT strain than in the Δ*cdgA*, Δ*cdgL*, Δ*cdgO*, Δ*cdgLO*, and Δ*cdgALO* strains (Fig 6B). These results reveal that these three DGCs have a hierarchical effect on *vps* expression, with CdgA having the largest effect and CdgO the least. We next evaluated the impact of these three DGCs on cellular c-di-GMP levels using the c-di-GMP fluorescence reporter. Abundance of c-di-GMP was higher in the WT strain than in the Δ*cdgA*, Δ*cdgL*, and Δ*cdgO* strains; while deletion of multiple DGCs in tandem (Δ*cdgLO* and Δ*cdgALO* strains) lowered c-di-GMP levels compared to the strains with single deletions (Fig 6C). This finding suggests that the relative contribution of the DGCs to c-di-GMP accumulation correlates with their contributions to *vps*-II expression (CdgA>CdgL>CdgO).

We next analyzed the contribution of CdgA, CdgL and CdgO to *vps*-II expression and c-di-GMP levels in the Δ*flaA* genetic background. Expression of *vps*-II was higher in the Δ*flaA* strain compared to the Δ*flaA*Δ*cdgA*, Δ*flaA*Δ*cdgL*, Δ*flaA*Δ*cdgO*, Δ*flaA*Δ*cdgLO*, and Δ*flaA*Δ*cdgALO* strains (Fig 6D). Thus, CdgA, CdgL, and CdgO regulate *vps*-II expression in the Δ*flaA* background (Fig 6D). The levels of c-di-GMP were also higher in the Δ*flaA* strain than in the Δ*flaA*Δ*cdgA*, Δ*flaA*Δ*cdgL*, Δ*flaA*Δ*cdgO*, Δ*flaA*Δ*cdgLO*, and Δ*flaA*Δ*cdgALO* strains (Fig 6E). Collectively, these results show that while the lack of all three DGCs significantly reduces the increase in c-di-GMP accumulation seen in the Δ*flaA* background, their individual contributions are minimal; notably, c-di-GMP level in the Δ*cdgALO* strain is lower than in the Δ*flaA*Δ*cdgALO* strain, suggesting that additional DGCs or PDEs also contribute to the c-di-GMP increase in the Δ*flaA* strain.

We further analyzed the c-di-GMP-accumulation profile of the Δ*cdgALO* and Δ*flaA*Δ*cdgALO* strains at early stages of biofilm formation in flow cells (Fig 6F and S1 Fig). The Δ*cdgALO* strain showed reduced c-di-GMP levels compared to the WT strain throughout the time course (Fig 6F and S1 Fig). The Δ*flaA*Δ*cdgALO* strain had lower c-di-GMP levels than the Δ*flaA* strain but higher than the Δ*cdgALO* (Fig 6F and S1 Fig). These results further support the model that c-di-GMP signaling modules different from CdgALO promote c-di-GMP accumulation in the absence of *flaA*. In addition, we found that c-di-GMP accumulation dynamics in the Δ*flaA*Δ*cdgALO* strain differs significantly from that of the Δ*flaA*Δ*motX* strain (Fig 6F and S1 Fig), suggesting that additional c-di-GMP signaling modules contribute to the stator-mediated modulation of c-di-GMP levels.

CdgA, CdgL and CdgO have predicted transmembrane domains. We speculated that these DGCs involved in the FDBR response could be localized to the flagellar pole either constitutively or in response to the absence of the flagellum filament. To test this, we chromosomally expressed HubP-sfGFP (superfolder green fluorescent protein), CdgA-sfGFP, CdgL-sfGFP, and CdgO-sfGFP (S1 Text). The positive control HubP-sfGFP localized to the cell poles as anticipated [63]. However, none of the DGCs localized to the cell poles in the WT strain or in the Δ*flaA* strain under the conditions tested (S3 Fig).

Our studies also identified the PDE *rocS* as a negative regulator of colony corrugation. RocS is a dual domain GGDEF and EAL protein that functions predominantly as a PDE [45,64]. We reasoned that RocS may be a key PDE keeping c-di-GMP levels low in a flagellar assembly/motor activity-dependent manner. To evaluate if RocS was the main c-di-GMP gatekeeper controlling colony corrugation in the Δ*flaA* strain, we generated the Δ*rocS* and Δ*flaA*Δ*rocS* strains and analyzed their colony morphologies. We found that the Δ*rocS* strain is more corrugated than the Δ*flaA* strain (S4 Fig). Although we cannot rule out the role of RocS as a gatekeeper of c-di-GMP levels during the FDBR response, the additive effect on colony corrugation observed in the Δ*flaA*Δ*rocS* strain suggests that other c-di-GMP gatekeepers might be involved in this process.

### Different flagellar mutants can trigger FDBR responses of varying magnitudes

To further characterize the FDBR response and identify potential signaling proteins involved, we designed a genetic screen to identify extragenic suppressors that regain the ability to form corrugated colonies in a Δ*flaA*Δ*motX* genetic background. Most of the suppressors we identified sustained insertions into genes encoding flagellar regulators of class I and class II (*flrA*, *flrB* and *flrC*) proteins that belong to the flagellum-specific transport machinery (*flhA*, *flhB*, *fliI*, *fliO*, *fliP* and *fliR*), to the MS ring (*fliF*) or C rings (*fliG* and *fliM*), or to the rod (*fliE* and *flgF*) (S2 Table). Most of these mutants are expected to affect the structure of the basal body, and most likely stator occupancy at the rotor [22]. Thus, the absence of flagellum components other than the filament can also promote the FDBR phenotype.

**Fig 7:**
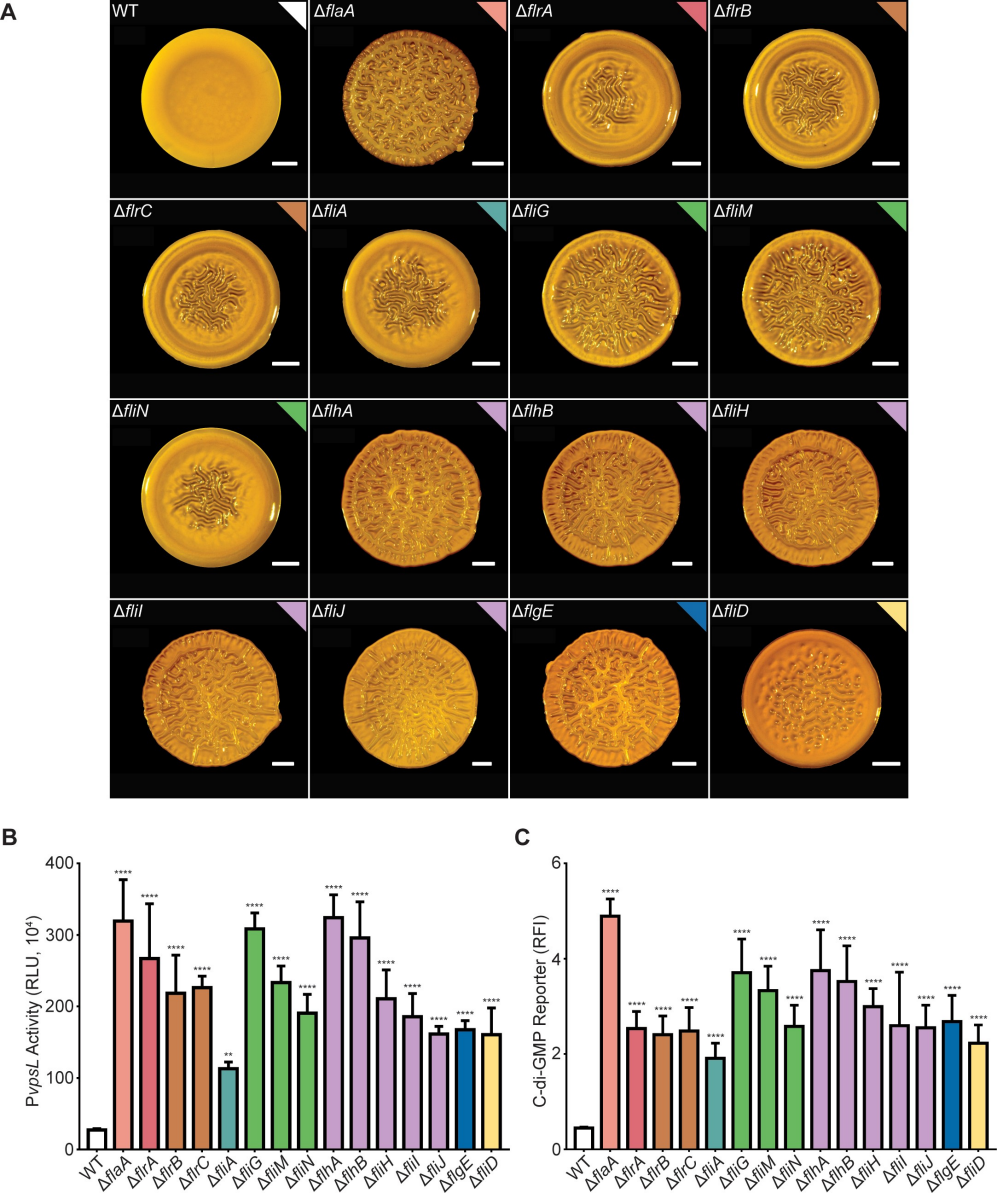
Strains lacking flagellum regulators or flagellum components have an FDBR response. A) Representative images of the colony morphologies of the WT strain and strains lacking a variety of flagellum regulators and flagellum components (some of these images are also presented in S5 Fig). Scale bars = 1 mm. B) Bar graph of means and standard deviations of RLU obtained from the transcription of *vpsL*-*luxCDABE* in colonies of the WT and flagellar mutant strains. C) Bar graph of means and standard deviations of RFI obtained from the expression of the c-di-GMP biosensor in colonies of the WT and flagellar mutant strains. Means obtained from 3 biological replicates were compared to WT with a one-way ANOVA followed by Dunnett’s multiple-comparison test. Adjusted P values ≤ 0.05 were deemed significant. ** p ≤ 0.01; **** p ≤ 0.0001. The color of each bar represents the type of flagellum structure to which each gene product belongs as depicted in Fig 1A.

To validate these results and further evaluate the ability of other flagellar mutants to trigger the FDBR response, we generated in-frame deletions of genes encoding the flagellar regulators (*flrA*, *flrB*, *flrC*, and *fliA*), components of the C-ring (*fliG*, *fliM*, and *fliN*), the flagellar T3SS (*flhA*, *flhB*, *fliI*, *fliH*, and *fliJ*), the flagellar hook (*flgE*), and the capping protein (*fliD*) in both the WT and Δ*motX* backgrounds (Fig 1A). In the WT background, single flagellar gene mutants demonstrated varying levels of colony corrugation (Fig 7A), and all mutants demonstrated increased *vps*-II operon expression and c-di-GMP levels compared to WT (Fig 7B and 7C), validating the presence of an FDBR response within these mutants. The magnitude of FDBR responses in the mutant strains were either reduced (Δ*flrA*, Δ*flrB*, Δ*flrC*, Δ*fliA*, Δ*fliN* and Δ*fliD*), intermediate (Δ*fliH*, Δ*fliI*, Δ*fliJ*, and Δ*flgE*), or comparable (Δ*fliG*, Δ*fliM*, Δ*flhA*, Δ*flhB*) to the FDBR response observed for the Δ*flaA* strain (Fig 7). Together these results suggest that alterations in different components of the flagellum influence the c-di-GMP-signaling modules that promote biofilm formation. The varied magnitude of responses within these strains, likely stem from differences in their abilities to alter the assembly of the flagellum rotor and/or stator.

**Fig 8:**
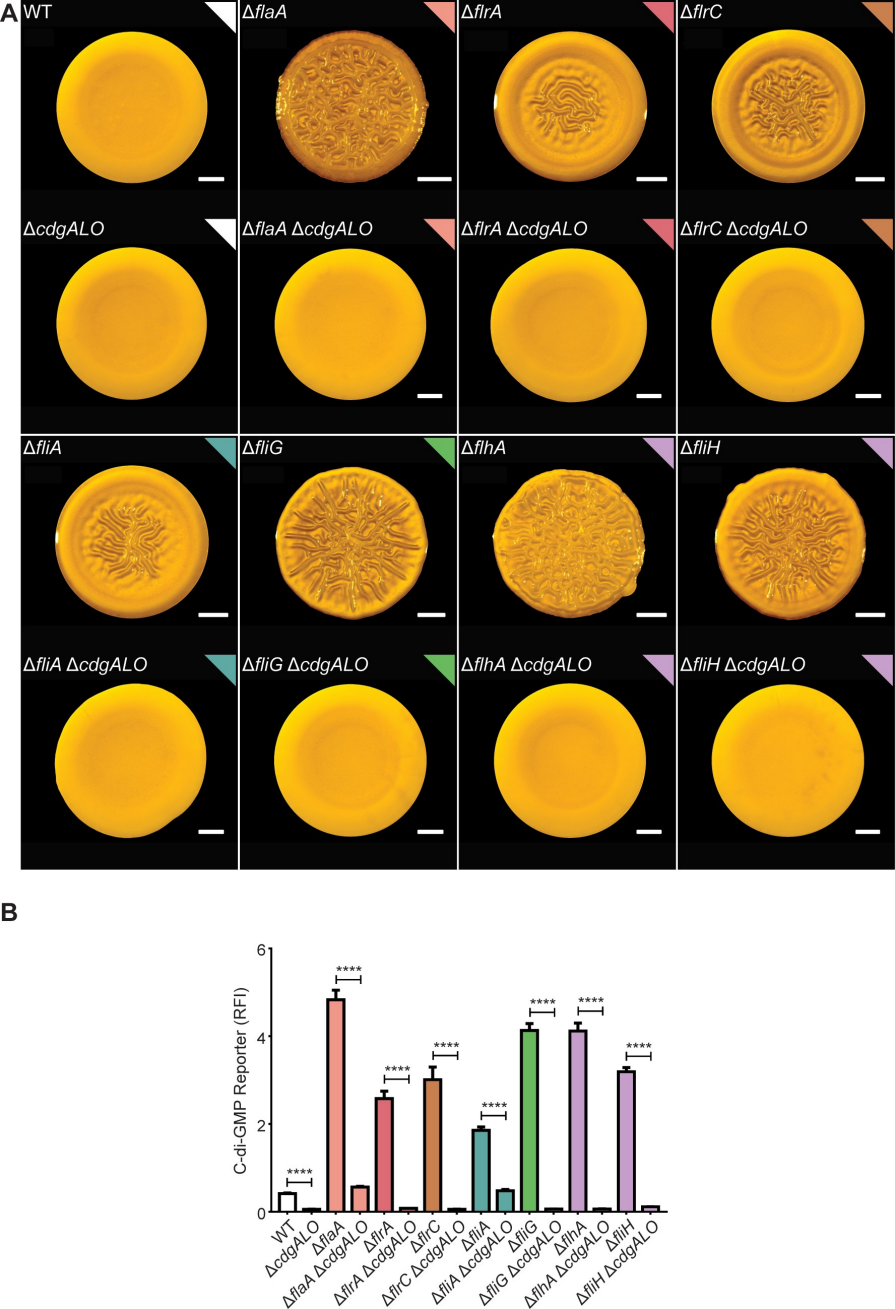
CdgA, CdgL, and CdgO are required for FDBR responses in flagellar mutants. A) Representative images of the colony morphologies of the WT strain and strains with null mutations in flagellar genes and the *cdgA*, *cdgL*, and *cdgO* genes. Scale bars = 1 mm. B) Bar graph of means and standard deviations of RFI obtained from the expression of the c-di-GMP biosensor in colonies of the strains indicated. Means obtained from 3 biological replicates were compared with an unpaired t-test. Each flagellar gene mutated is color coded accordingly to its function as indicated in the illustration in Fig 1A.

We next analyzed whether the DGCs CdgA, CdgL, and CdgO are also necessary for the FDBR responses in flagellar mutants other than Δ*flaA*. To test this, we generated quadruple deletions lacking a representative flagellar gene as well as *cdgA*, *cdgL*, and *cdgO*. In all these quadruple mutants, colony corrugation was lost (Fig 8A). Furthermore, c-di-GMP accumulation did not occur or was significantly impaired in the quadruple mutants compared to the corresponding single-deletion mutant in the flagellar gene (Fig 8B). The Δ*flaA*Δ*cdgALO* and the Δ*fliA*Δ*cdgALO* strains had c-di-GMP levels that were 9.7- and 8.3-fold higher, respectively, compared to the Δ*cdgALO* strain. In contrast, the rest of the quadruple mutants showed only a 2-fold increase (Δ*fliH*Δ*cdgALO*) or the same c-di-GMP levels compared to Δ*cdgALO*. These findings suggest that the extent of the requirement for CdgA, CdgL and CdgO varies among the different flagellar mutants analyzed.

### Different flagellar mutants show differences in stator-mediated FDBR responses

To determine stator impacts on FDBR responses within these flagellar gene deletions, we next generated double mutants combining the flagellar gene mutations with deletion of *motX*. The FDBR phenotype observed in Δ*flrA* and Δ*flrBC* strains was not dependent on the presence of MotX, whereas in Δ*fliA* strain it was (S5 Fig). Given that FliA regulates the expression of *motX*, the observed MotX-dependent FDBR response in the Δ*fliA* strain was unexpected. We therefore analyzed the expression of a *motX*-*luxCDABE* transcriptional fusion in the WT, Δ*flrA* (class I regulator), and Δ*fliA* (class IV regulator) strains. Expression of *motX* was markedly reduced, but not completely eliminated in the Δ*fliA* strain (S6 Fig). As a positive control, expression of the class I gene *flaA* (*flaA*-*luxCDABE*) was exclusively and completely dependent on FlrA (S6 Fig), as expected. This finding suggests that expression of *motX* is not fully dependent on FliA, and perhaps could explain the effect of the absence of *motX* on the Δ*fliA* FDBR response.

Collectively, our findings indicate that suppression of the FDBR response by the lack of MotX lays within a continuum (S5 Fig): at one end are strains lacking basal body components (FliG, FliM, FliN, FlhA, FlhB) and the regulators responsible for their production (FlrA and FlrBC) that showed an FDBR response insensitive to the absence of MotX; in the middle are strains including those lacking flagellar axial components (*flgE*) or the flagellum ATPase complex (FliI and FliH) that showed an intermediate FDBR phenotype in the absence of MotX; and at the other end are strains that showed an FDBR response that was fully sensitive to the absence of MotX, including strains lacking the flagellum filament (FlaA and FliD) and the class IV regulator FliA (S5 Fig).

### The FDBR response cannot be solely triggered by VpsR^D59E^ or absence of HapR

**Fig 9:**
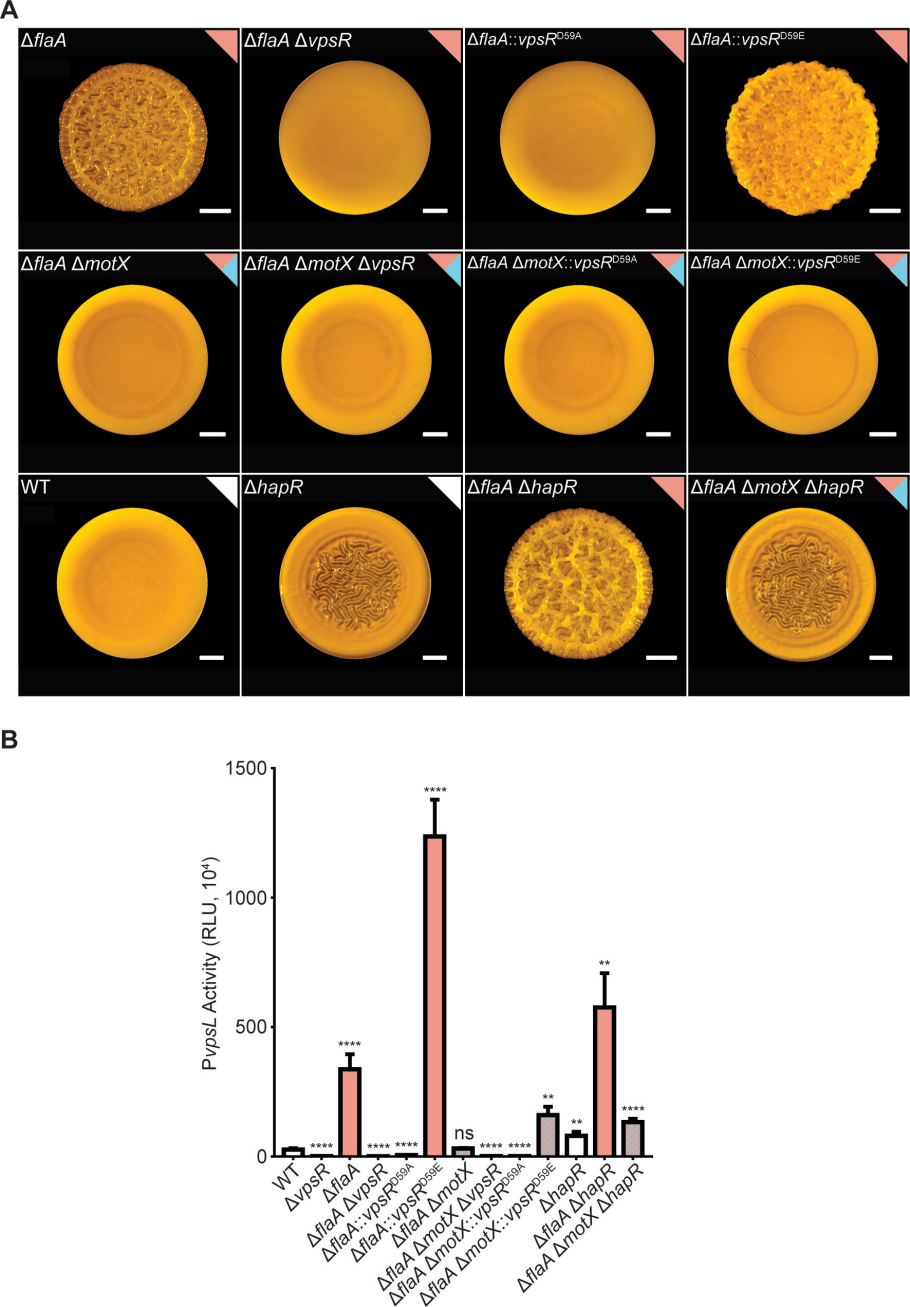
VpsR^D59E^ or absence of HapR do not promote colony corrugation or increased *vps*-II expression in the Δ*flaA* Δ*motX* strain to the levels observed in the Δ*flaA* strain. A) Representative images of colony morphologies of indicated genetic backgrounds. B) Bar graph of means and standard deviations of RLU obtained from the transcription of *vpsL*-*luxCDABE* in colonies. Means obtained from at least three independent biological replicates were transformed to adjust for unequal standard deviations and compared to the WT strain with a one-way ANOVA and Dunnett’s multiple-comparison test. Adjusted P values ≤ 0.05 were deemed significant. **** p ≤ 0.0001, ns not significant.

The main activator of *vps* gene expression and biofilm formation is the transcriptional activator VpsR, a response regulator [39,44,46]. Production of VpsR is controlled by c-di-GMP levels, and it has been proposed that its activity is also regulated by c-di-GMP post-translationally [41,44,53]. There is also indirect evidence that the VpsR phosphorylation state regulates its activity [51,65]. To determine the extent of involvement of VpsR and its phosphorylation on *vps*-II expression during the FDBR response, we generated Δ*flaA* and Δ*flaA*Δ*motX* strains lacking *vpsR* or producing inactive (*vpsR*^D59A^) and overactive variants (*vpsR*^D59E^) of VpsR with point mutations in its receiver domain. The Δ*flaA*Δ*vpsR and* Δ*flaA*Δ*vpsR*:: *vpsR*^D59A^ strains showed a smooth colony morphology and did not express the *vps*-II operon (Fig 9). In contrast, the colonies of the Δ*flaA*::*vpsR*^D59E^ strain showed enhanced corrugation compared to the Δ*flaA* strain, and higher expression of the *vps*-II operon compared to the WT or the Δ*flaA* strains (Fig 9). These results indicate that VpsR is required for the FDBR response and that activation of VpsR can potentiate the FDBR response of the Δ*flaA* strain.

We next evaluated if the production of the overactive VpsR^D59E^ variant could promote colony corrugation and *vps*-II expression in the Δ*flaA*Δ*motX* strain. The colonies of the Δ*flaA*Δ*motX*Δ*vpsR*::*vpsR*^D59E^ strain were smooth although more compact compared to those of the Δ*flaA*Δ*motX*, Δ*flaA*Δ*motX*Δ*vpsR* and Δ*flaA*Δ*motX*Δ*vpsR*::*vpsR*^D59A^ strains (Fig 9A). Expression of the *vps*-II operon was 5.6-fold higher in the Δ*flaA*Δ*motX*Δ*vpsR*::*vpsR*^D59E^ strain compared to the WT strain (Fig 9B). These results suggest that the level of induction of *vps* genes observed in the Δ*flaA*Δ*motX*Δ*vpsR*::*vpsR*^D59E^ strain is not sufficient to promote colony corrugation. Production of a VpsR^D59E^ variant cannot rescue the FDBR response in the Δ*flaA*Δ*motX* strain, further suggesting that the c-di-GMP increase is required for a complete activation of the FDBR response.

In *V*. *cholerae*, abundance of HapR, the master regulator of quorum-sensing, is positively regulated by the quorum-sensing signaling module and negatively regulated by FliA [66,67]. We speculated that corrugation in the Δ*flaA* strain could be due to reduced HapR levels. Colonies of the Δ*hapR* strain are less corrugated than colonies of the Δ*flaA* strain (Fig 9A). This implies that HapR is not the dominant regulator of the FDBR response. Furthermore, colonies of the Δ*flaA*Δ*hapR* strain were more corrugated than the Δ*flaA* and Δ*hapR* strains, and colonies of the Δ*flaA*Δ*motX*Δ*hapR* strain were visually identical to colonies of the Δ*hapR* strain. These results suggest that the biofilm phenotypes associated with FlaA, MotX and HapR are not interdependent. We additionally analyzed expression of *vps*-II in these strains and found that the pattern of expression of this promoter correlates with the observed colony morphologies (Fig 9B). The absence of *hapR* induced *vps*-II expression but not to the levels observed in the absence of *flaA*. These results are suggestive of independent regulatory roles of FlaA and HapR; however, with the current evidence we cannot rule out a potential interconnection between the c-di-GMP signaling modules associated with the assembly of the flagellum filament and those associated with the presence of an active HapR.

## Discussion

**Fig 10:**
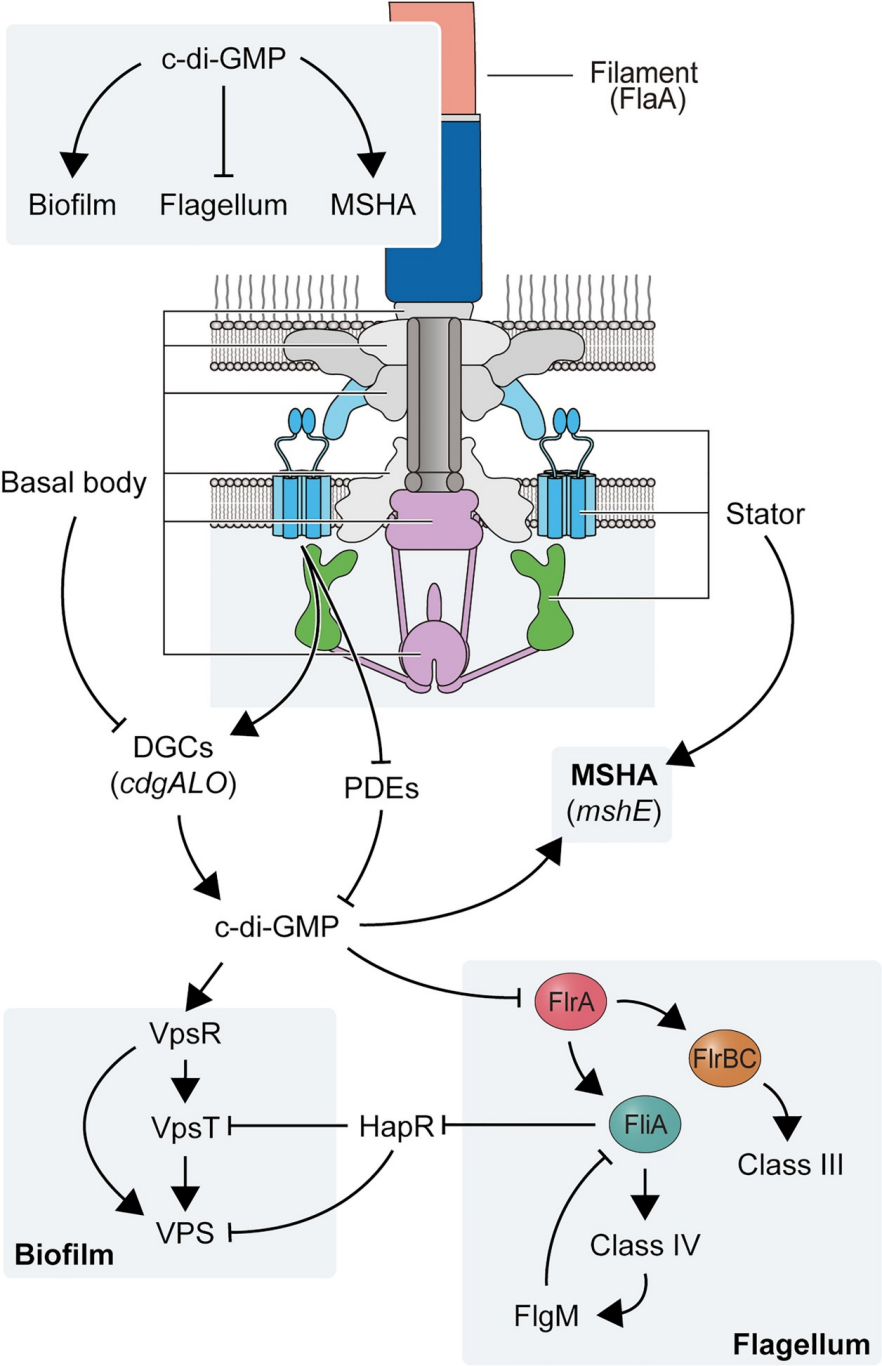
A model for signal transduction during the FDBR response in *V*. *cholerae*. Illustration showing the connection between main components of the polar flagellum and processes regulated by c-di-GMP signaling. Lines ending in arrows indicate positive regulation and lines ending with a perpendicular line indicate negative regulation.

Regulation of flagellar motility is an important aspect of biofilm formation. At the early stages of biofilm formation, it is predicted that functional inhibition (flagellar rotation) of the flagellum is necessary to stabilize cell-surface attachment, preventing detachment. The second messenger c-di-GMP is at the core of the regulatory circuits that control motility and biofilm formation: High levels of c-di-GMP repress flagellar production and activity. In this study, we observed that *V*. *cholerae* cells lacking components of the flagellum differ in biofilm gene expression, biofilm formation, and cellular concentrations of c-di-GMP (FDBR response) compared to the WT strain (Fig 10). The lack of flagellar components such as the basal body and flagellar axial proteins promote biofilm formation and c-di-GMP accumulation. In contrast, the flagellum stator is needed to activate biofilm formation in WT strains and in mutants lacking axial proteins such as FlaA and FliD. The presence of a stator that cannot bind Na^+^ as well as the absence of the sodium pumping Na^+^-NQR complex suppresses biofilm formation in the Δ*flaA* strain. The exact identity of the signal transduced through the flagellum stator to control biofilm formation is not yet known. The absence of the stator and/or the Na^+^-NQR complex could alter Na^+^ homeostasis and, in turn, the Na^+^ motive force and the membrane potential. The dynamics of stator occupancy around the flagellum rotor of *V*. *alginolyticus* depend on both the concentration of Na^+^ and the Na^+^ motive force [20,60]. It is possible that in *V*. *cholerae* the load perceived by the flagellum motor decreases in the absence of FlaA, this would in turn result in lower stator occupancy at the rotor, and in altered ion homeostasis. The mechanisms by which the cell can perceive changes in stator occupancy are being investigated in other organisms and have been linked to c-di-GMP signaling [57,68].

c-di-GMP is central to surface sensing mediated by different cell-surface structures. In *Caulobacter crescentus*, c-di-GMP signaling activates a single DGC DgcB to mediate a tactile response that is transduced through the flagellum motor [68]. *Pseudomonas aeruginosa* switches from one type of flagellum stator to another (MotAB and MotCD) depending on the flagellum load in a process that involves changes in c-di-GMP levels [57]. Absence of both stators results in decreased c-di-GMP accumulation compared to the WT strain [69]. This process is regulated through the interaction of MotC with the DGC SadC, which results in activation of the latter [69]. In *V*. *cholerae*, no single deletion of any of the 28 conserved DGCs encoded in its genome fully suppressed the FDBR response of the Δ*flaA* strain. The DGC CdgF from *V*. *cholerae* has 47.5% similarity to DgcB from *C*. *crescentus*; however, the absence of this DGC did not significantly affect colony corrugation in a Δ*flaA* genetic background. No orthologue of SadC is encoded in the genome of *V*. *cholerae*. We identified three DGCs (CdgA, CdgL, and CdgO) that are required for the FDBR response. These three DGCs do not localize to the flagellar pole and might not be specific for the FDBR response, but they are clearly crucial for signaling cascades that trigger enhanced biofilm matrix production. We also entertained the possibility that the FDBR response could be triggered by reduced abundance or activity of a “flagellum-associated” PDE. Our finding that a transposon insertion in *rocS* (PDE) can promote FDBR response in the Δ*flaA*Δ*motX* strain led us to evaluate if *rocS* and *flaA* were in the same pathway. We found that that the lack of FlaA and RocS has an additive effect in FDBR, suggesting that other c-di-GMP gatekeepers, could be downregulated in the absence of FlaA.

Our model is that the lack of the flagellum filament generates a signal that is transduced by functional flagellar stators and results in elevated c-di-GMP levels and biofilm formation. Regardless of the presence or absence of the flagellum filament, functional stators appear to be crucial to maintain c-di-GMP levels during initial stages of surface colonization and to enable surface attachment. The absence of the flagellum stator severely compromises surface attachment, lowers c-di-GMP levels, and lowers MSHA production compared to the WT strain (Fig 10). Since the activity of the ATPase MshE is positively regulated by c-di-GMP [27,28,70], it is possible that the regulation of MSHA pili abundance by the flagellum stator is at the level of MshE activation. Our results suggest that the FDBR response requires the input from the DGCs CdgA, CdgL and CdgO, however it is unknown if these same DGCs participate in the activation of MshE. A clear example of interconnectivity between appendages during a tactile response comes from the Tad pili and the flagellum motor of *C*. *crescentus* [71]. In this bacterium, the Tad pili positions the flagellum motor in a way that facilitates permanent adhesion. The tactile response of this bacterium is mediated by c-di-GMP through affecting the dynamics of pilus retraction and activating holdfast synthesis in a motor-dependent mechanism [68,71]. The coordinated assembly of surface appendages is a process that has not been explored in great depth in *V*. *cholerae* and could be of major significance for the adaptability of this pathogen during the colonization of diverse niches.

In our model, the initial cellular c-di-GMP increase following surface attachment likely activates the VpsR-VpsT c-di-GMP effectors through signaling modules that employ CdgA, CdgL, and CdgO [44,46], which further increases c-di-GMP levels, VpsT activation and induction of *vps* gene expression [62] (Fig 10). The continuous buildup of c-di-GMP concentrations could allosterically inactivate FlrA and downregulate flagellar gene expression [16]. Lack of FlrA activity could trigger a FDBR response, which could be a mechanism to maintain elevated biofilm matrix production during biofilm formation. We also showed that a mutation that mimics constitutive phosphorylation of VpsR did not rescue defects in the FDBR response in the Δ*flaA*Δ*motX* strain. This implies that post-translational modifications at D59 are not the main mechanism triggering FDBR. Nonetheless, VpsR activation, most likely through c-di-GMP, is key for increased biofilm matrix production during the FDBR response. We also analyzed the role of HapR, a direct repressor of *vps* genes and *cdgA*, in the FDBR response [43,44,72]. We found that strains lacking both HapR and FlaA exhibit enhanced *vps* expression and biofilm formation, suggesting that they act through different pathways. HapR abundance is negatively regulated by FliA [67]; hence, c-di-GMP-dependent inactivation of FlrA and subsequent downregulation of *fliA* could result in de-repression of *hapR* at later stages of biofilm formation. Furthermore, quorum sensing at high cell density promotes expression of PDEs, including *rocS*, most likely through HapR signaling [43,44]. We therefore propose that in mature biofilms, HapR production and activation of the HapR regulon would lower c-di-GMP levels and promote biofilm dispersal.

In summary, our findings suggest that proper flagellum assembly and flagellar function limits c-di-GMP accumulation, thereby favoring motility over surface commitment and biofilm formation. During its infection cycle, *V*. *cholerae* experiences stochastic and regulated flagellar breaks. For example, mucosal penetration during colonization of intestinal epithelial cells leads to flagellum breaks; this process initiates virulence factor production [67]. Some γ-proteobacteria, including *V*. *cholerae*, eject their flagellum under nutrient-depleted conditions [73]. During biofilm formation, surface attachment and mechanical forces operating in biofilms could result in flagellum breaks and in turn generation of a heterogeneous population of flagellated and non-flagellated cells with different levels of c-di-GMP. This in turn would lead to differences in matrix production and altered architecture and stratification of biofilms. Environmental conditions that favor c-di-GMP accumulation could result in reduced flagellar gene expression due to the allosteric inhibition of FlrA. Reduced flagellar gene expression could potentially trigger an FDBR response that enable full commitment towards biofilm formation. Our work reveals the connection between flagellum assembly, production of cell surface appendages, biofilm matrix production, and c-di-GMP signaling. This study also reveals key aspects of a biological phenomenon that exemplifies the complexities of the decision-making processes of *V*. *cholerae* and improves our knowledge of the behavior of this important human pathogen.

## Materials and methods

### Strains and growth conditions

The strains used are listed in S3 Table. Bacterial cultures were grown in lysogeny broth (LB) (ddH_2_O, 1% NaCl (w/v), 1% tryptone, 0.5% yeast extract, pH 7.5) at 30°C with aeration (200 rpm). Colony biofilms were grown in LB agar plates (1.5% Bacto-Agar). Antibiotics were added to cultures of *V*. *cholerae* containing plasmids at the following concentrations: 5 μg/mL chloramphenicol or 100 μg/mL ampicillin or 100 μg/mL streptomycin. Cultures of *Escherichia coli* containing plasmids were grown in the presence of 20 μg/mL chloramphenicol or 100 μg/mL ampicillin.

### Recombinant DNA techniques and genetic manipulation

DNA manipulations were performed using standard molecular techniques. The high-fidelity DNA polymerase Q5 (New England Biolabs) was used for PCR amplification. Primers were designed using the NEBuilder Assembly Tool or the NEBaseChanger tool and synthesized by Integrated DNA Technologies. DNA cloning was performed by isothermal assembly (Gibson assembly) using NEBuilder HiFi DNA Assembly Master Mix (New England Biolabs). To generate deletion constructs, two DNA fragments of approximately 500 bp containing the truncated gene and upstream and downstream sequence were assembled into the suicide plasmid pGP704sac28. Constructs made to knock-in variants with specific point mutations or to add a C-terminal superfolder GFP (5xGly-sfGFP) tag were also cloned into pGP704sac28. The wild-type version of the gene of interest plus 500-bp upstream and 500-bp downstream was assembled into pGP704sac28. Point mutations were generated using the Q5 site directed mutagenesis kit (New England Biolabs). The constructs used to insert the 5xGly-sfGFP tag contained approximately 500-bp upstream of the stop codon of the gene of interest and 500-bp downstream of the stop codon. The native stop codon was removed, and sequence encoding five glycine residues in tandem was added instead. The sfGFP sequence was amplified from plasmid pFY_5676. The transcriptional fusion of the regulatory region of *vpsL* and the *luxCDABE* operon was assembled in the plasmid pBBR*lux*. The regulatory region of *vpsL* was amplified from genomic DNA of the C6706 strain.

Plasmids were mobilized by biparental mating using the donor *E*. *coli* SM10λ*pir* strain. Briefly, cultures of the donor and recipient strains were mixed 1:1, and mating spots were grown on LB agar plates (37°C, 6 h). Transconjugants were selected on LB agar plates containing streptomycin (100 μg/mL) and chloramphenicol (5 μg/mL) or ampicillin (100 μg/mL). Genetic knock-out and knock-in procedures were performed as previously specified [45].

### Analysis of colony morphology

Colony biofilms grown for qualitative analysis were made from cultures inoculated with five single colonies grown overnight at 30°C with aeration (200 rpm). Cultures were diluted 1:200 in LB, and 2 μL were spotted in technical triplicates on Petri dishes containing 20 mL of LB agar. Once the spots were dry, the plate was incubated at 30°C for 24 h and imaged using a Zeiss stereo microscope coupled with an Axiocam ERc 5s camera.

### Luminescence assay

Colony biofilms of *V*. *cholerae* strains harboring the P*vpsL*-*lux* construct, were grown for 24 h at 30°C on LB agar plates containing chloramphenicol (5 μg/mL). Individual spots were scraped using a 10 μL loop, transferred to 1 mL of LB containing sterile glass beads, and vortexed. A 200-μL aliquot of the suspension was added to a white, flat-bottom 96-well plate in triplicate (technical duplicate spots were used). Luminescence and optical density (600 nm) were measured using a Perkin Elmer Victor3 multilabel counter. Relative luminescence units (RLU) are expressed as luminescent counts · min^−1^ · mL^−1^ · OD_600_^−1^. Assays were performed in three independent biological replicates. Statistical analysis was performed using GraphPad Prism 7.

### Analysis of c-di-GMP abundance

Colony biofilms of *V*. *cholerae* strains were grown for 24 h at 30°C on LB agar plates. Intracellular c-di-GMP quantification via mass spectroscopy was done for a given strain from 20 spot biofilms. The spots were pooled in 1 mL LB, containing sterile glass beads and vortexed. After being spun down, decanted, and resuspended with 2.5 mL of 2% SDS, 250 μL was removed and used for BCA quantification. The remaining 750 μL of suspension was spun down, decanted, and resuspended in 1 mL of extraction buffer (40% acetonitrile, 40% methanol, 0.1% formic acid, 19.9% HPLC grade H_2_O). Insoluble components were spun down, and 800 μL of the supernatant was collected and dried under vacuum. The dried sample was then resuspended in 50 μL HPLC grade H_2_O containing 184 mM NaCl, and c-di-GMP was quantified via LC-MS/MS at the UCSC Chemistry and Biochemistry Mass Spectrometry facility. c-di-GMP standard curves were generated using c-di-GMP standards (SIGMA) of 25, 50, 100, 500, 2000, 3500, and 5000 nM dissolved in HPLC grade H_2_O containing 184 mM NaCl. The abundance of c-di-GMP was extrapolated from the mass spectroscopy data and normalized to protein abundance per 1 mL of the spot suspension. Intracellular levels of c-di-GMP were evaluated using a fluorescent reporter as previously described [74,75]. In brief, spot biofilms of *V*. *cholerae* strains harboring the pMMB67EH-Bc3-5 biosensor were grown for 24 h at 30°C on LB agar plates containing ampicillin (100 μg/mL). Individual spots were scraped using a 10 μL loop, transferred to 1 mL of LB containing sterile glass beads, and vortexed. An aliquot of 200 μl of the cell suspension was transferred to Corning 96-well, clear-bottom, black, polystyrene microplates, and fluorescence was measured in a Victor X3 plate reader (PerkinElmer). Excitation/emission filters of 460/480 nm and 550/580 nm for Amcyan and TurboRFP, respectively, were used to measure fluorescence intensity. The background fluorescence obtained from a strain harboring the empty plasmid pMMB67EH was subtracted from fluorescence of the experimental samples. The relative fluorescence intensity (RFI) were calculated from the ratio of fluorescence intensity of TurboRFP to Amcyan.

### Biofilm competition assays

Overnight cultures of WT::Tn7_RFP (WT-RFP), WT::Tn7_GFP (WT-GFP), and mutant::Tn7_GFP (Δ*flaA*, Δ*motX*, and Δ*flaA* Δ*motX* mutants) were inoculated into 5 mL of LB media from five single colonies and incubated at 30°C with 200 rpm shaking overnight (~14–18 h). WT-RFP and either WT-GFP or mutant-GFP strains were then mixed, each at a 1:400 dilution, in 1 mL of 2% LB media, and 200 μL of mixtures were pipetted into channels of an μ-Slide VI 0.4 uncoated, plastic-bottom slide (Ibidi), and cells were allowed to attach for 1 h at room-temperature. Following attachment, flow of 2% LB media was established at a rate of ~8 mL per channel per h, and biofilms were allowed to form at room temperature. Images of the developing biomass were obtained on a Zeiss LSM 880 confocal microscope at 1, 3, 6, and 24 h post establishment of flow at 20x magnification for biomass analysis and 40x magnification for image generation. Images were processed with Imaris (Oxford Instruments), and biomass quantification was performed using COMSTAT2 [76,77].

### Quantification of single-cell c-di-GMP relative abundances in flow cells using a biosensor

Flow cells were prepared and inoculated as previously described [74,78]. Cultures for flow cells were prepared as previously described [74] with the following modifications. The diluted bacteria culture (taken from an overnight liquid culture) was injected into the flow cell and allowed to incubate for 10–60 min without flow on the heating stage at 30°C for cells to adhere to the surface. This variable incubation time without flow allowed strains with lower attachment to start the experiment with a similar number of cells in the field of view compared to strains without attachment defects. Flow was then started at 3 mL/h for the entire acquisition time. Time t = 0 h corresponded to when the image acquisition began after the flow started.

Images were taken as previously described [74,78] with the following modifications. Images were taken using an Andor iXon EMCCD camera with Andor IQ software on an Olympus IX81 microscope equipped with a Zero Drift Correction autofocus system. Bright-field and fluorescence images for the c-di-GMP biosensor were taken as previously described [74]. Image size was 67 μm × 67 μm (1024 × 1024 pixels). Image analysis and other related calculations (e.g., segmentation, RFI values) were performed in MATLAB as previously described [74]. The method for obtaining the distribution of RFI values is summarized as follows. In these experiments, each time point is analyzed independently. For each time point, which is a single image, the pixels belonging to bacteria on the surface are identified via segmentation using our previously described algorithm [74]. These pixel locations are then used to extract fluorescence intensities for both reporter and control and then divided to get RFI values. If multiple experiments are performed (for this manuscript, 2 independent experiments were performed per strain), then the RFI values for each corresponding time point and image are combined into a final distribution of RFI values. For each of these distributions per time point, the median was calculated, and then bootstrap sampling was performed to obtain a bootstrap sampling distribution of the median values. These bootstrap sampling distributions can then be used to obtain the 95% confidence intervals and directly compared to query for statistical significance.

### MSHA-specific hemagglutination assay

Surface MSHA pilus levels were determined by the ability of cells to hemagglutinate (HA) sheep erythrocytes. Briefly, cultures were inoculated with 5 single colonies from LB-agar plates into 5 mL of LB media, and incubated at 30°C with 200 rpm shaking for 14–18 hours. Cultures were diluted 1:200 into 5mL of fresh LB media, and incubated at 30°C with 200 rpm shaking until the OD_600_ was ~0.6–0.8. For each strain, cell numbers equivalent to OD_600_ of 0.4 per mL were pelleted at 4000 rpm for 10 minutes at 4°C, and washed twice with KRT buffer (10 mM Tris-HCl, pH 7.4; with 7.5 g NaCl, 0.383 g KCl, 0.318 g MgSO_4_.H_2_O, 0.305 g CaCl_2_ per liter) [79]. Finally, cells were resuspended in 1mL KRT buffer. For analysis of hemagglutination: 100 μL of this cell suspension was placed in the first column of a 96-well round-bottom plate, and 50 μL was then serially diluted down the remaining 11 columns which had been prefilled with 50 μL of KRT buffer (50 μL discarded from the final column). The first row of each plate was left blank with KRT buffer only as an untreated control. Erythrocytes from defibrinated sheep blood (Hardy Diagnostics) were resuspended on ice to a final concentration of 2% in KRT buffer. Erythrocytes were pelleted at 2000 rpm for 5 minutes at 4°C, and washed with KRT buffer until the supernatant was clear or a minimum of 2 washes. Then 50 μL of the 2% erythrocyte suspension was transferred to each well, and plates were covered and incubated at 4°C overnight. The HA titer was determined to be the lowest dilution containing visible signs of hemagglutination for each strain. Data is presented as the reciprocal of the lowest dilution with visible hemagglutination, and assays were performed in five independent biological replicates each with two technical replicates. Statistical analysis was performed using GraphPad Prism 8.

### Analysis of cell surface attachment

Strains were grown at 30°C with 200 rpm shaking until the OD_600_ was 0.4–0.6 in LB media. Strains were normalized to an OD_600_ of 0.02 in defined artificial seawater (DASW) [80], and 350 μL of each strain was added to the well of an μ-Slide 8 well uncoated plastic bottom microscopy slide (Ibidi GmbH). Slides were incubated statically for 1 hour at 30°C to allow for cell attachment. Supernatant was then removed, and non-adherent cells removed with two washes of 350 μL DASW. Cells were then visualized at 40x magnification on a Zeiss Axiovert 200 phase contrast microscope outfitted with a CoolSNAP HQ2 monochrome CCD camera (Photometrics). A total of 6 images were collected for each biological replicate of each strain, and 4 biological replicates were analyzed for each strain. Image J version Fiji 2.0.0-rc-69 was used to quantify surface attached cells. Images were inverted and the threshold was manually adjusted for each image to include only surface-attached cells in the analysis. Images were processed with a binary watershed to distinguish overlapping and dividing cells. Statistical analysis was performed using GraphPad Prism 8.

## Supporting information

S1 Fig Statistics for the c-di-GMP reporter flow cell assays. A) Measuring c-di-GMP levels of single cells inside a flow cell using the Bc3-5 biosensor. For each time point and strain, the distribution of RFI values were obtained from 2 independent experiments. Lines indicate the median RFI values per time point, and the shaded areas represent the 95% confidence intervals obtained from the bootstrap sampling distribution of the median RFI values. Time t = 0 h corresponds to when image acquisition began after flow started, rather than inoculation time. This allows a more unbiased comparison of surface attached cells between strains with and without attachment defects. B) Number of surface cells counted from the 2 independent experiments. The time axis is the same as in part A.
(TIF)

S2 Fig Individual deletions of the genes encoding the 28 conserved DGCs of *V*. *cholerae* have variable effects on colony corrugation in the Δ*flaA* background. Representative images of the colony morphologies of the WT and Δ*flaA* strains and double mutants lacking *flaA* and each individual DGC encoded in the genome of *V*. *cholerae*.
(TIF)

S3 Fig CdgA, CdgL, and CdgO are not localized to the cell pole. Representative bright-field and fluorescence microscopy images showing the intracellular distributions of superfolder GFP-labeled CdgA, CdgL, and CdgO in individual cells from the A) WT and B) Δ*flaA* genetic backgrounds. HubP was used as a positive control for polar localization. Scale bars = 5 μm.
(TIF)

S4 Fig FlaA regulates colony corrugation independently of RocS. Representative images of the colony morphologies of the WT, Δ*rocS*, Δ*flaA*, and Δ*flaA* Δ*rocS* strains.
(TIF)

S5 Fig The requirement for stator assembly to trigger the FDBR response is not universal among flagellar mutants. Representative images of the colony morphologies of the WT strain and a variety of flagellar mutants also lacking the T-ring gene *motX* (the same images of the single mutants are shown in Fig 7).
(TIF)

S6 Fig The *motX* gene is regulated by FlrA and FliA in *V*. *cholerae* O1 El Tor C6706. Bar graph of means and standard deviations of RLU obtained from the transcription of A) *flaA*-*luxCDABE* or B) *motX*-*luxCDABE* in exponentially grown cells. Means obtained from at least three independent biological replicates were compared to the WT strain with a one-way ANOVA and Dunnett’s multiple-comparison test. Adjusted P values ≤ 0.05 were deemed significant. *** p ≤ 0.001 **** p ≤ 0.0001.
(TIF)

S1 Table COMSTAT2 quantification values for flow cell biofilm competition model. (PDF)

S2 Table Transposon insertions that suppress biofilm matrix repression in Δ*flaA* Δ*motX*. (PDF)

S3 Table Table of strains and plasmids. (PDF)

S1 Text Extended methods. (PDF)

S1 Data Raw data used for all graphs and tables included in this work. (XLSX)
