# Supplementary figures and images for: Reciprocal c-di-GMP signaling: Incomplete flagellum biogenesis triggers c-di-GMP signaling pathways that promote biofilm formation
Source: PLoS Genet. 2020 Mar 16;16(3):e1008703. doi: 10.1371/journal.pgen.1008703 (PMC7098655; doi:10.1371/journal.pgen.1008703)

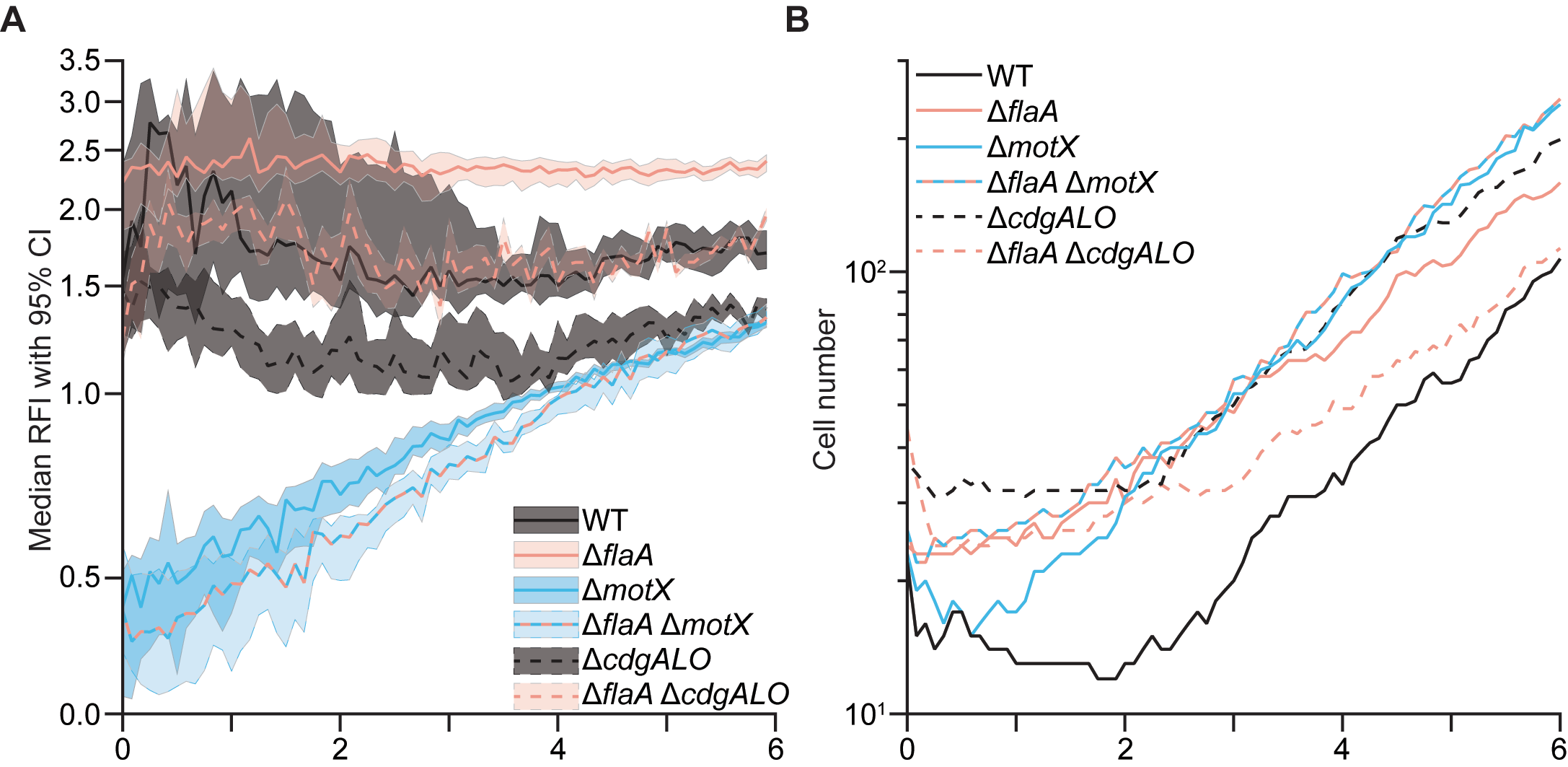

Supplement: S1 Fig — A) Measuring c-di-GMP levels of single cells inside a flow cell using the Bc3-5 biosensor. For each time point and strain, the distribution of RFI values were obtained from 2 independent experiments. Lines indicate the median RFI values per time point, and the shaded areas represent the 95% confidence intervals obtained from the bootstrap sampling distribution of the median RFI values. Time t = 0 h corresponds to when image acquisition began after flow started, rather than inoculation time. This allows a more unbiased comparison of surface attached cells between strains with and without attachment defects. B) Number of surface cells counted from the 2 independent experiments. The time axis is the same as in part A. (TIF) [file pgen.1008703.s001.tif]

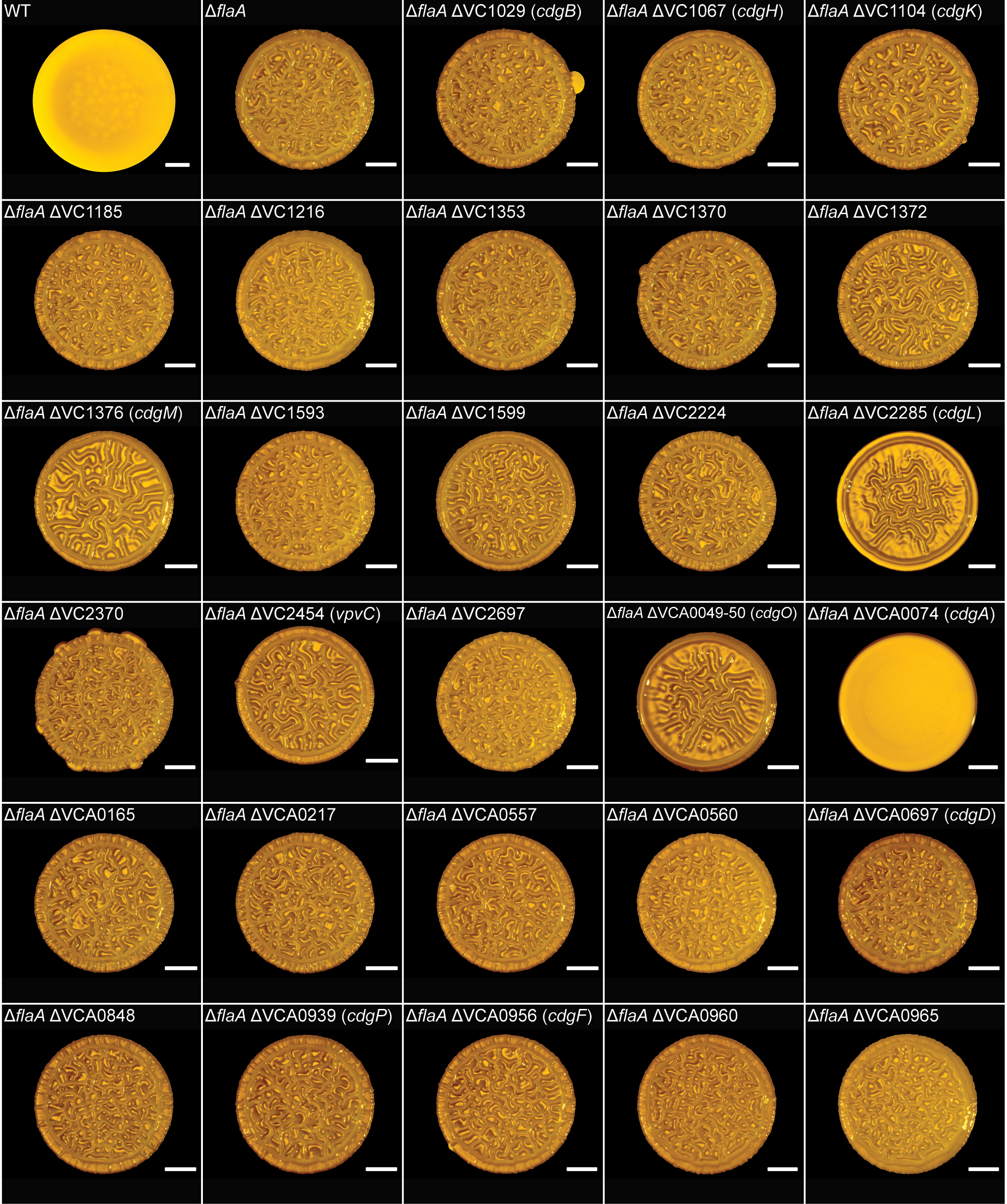

Supplement: S2 Fig — Representative images of the colony morphologies of the WT and ΔflaA strains and double mutants lacking flaA and each individual DGC encoded in the genome of V. cholerae. (TIF) [file pgen.1008703.s002.tif]

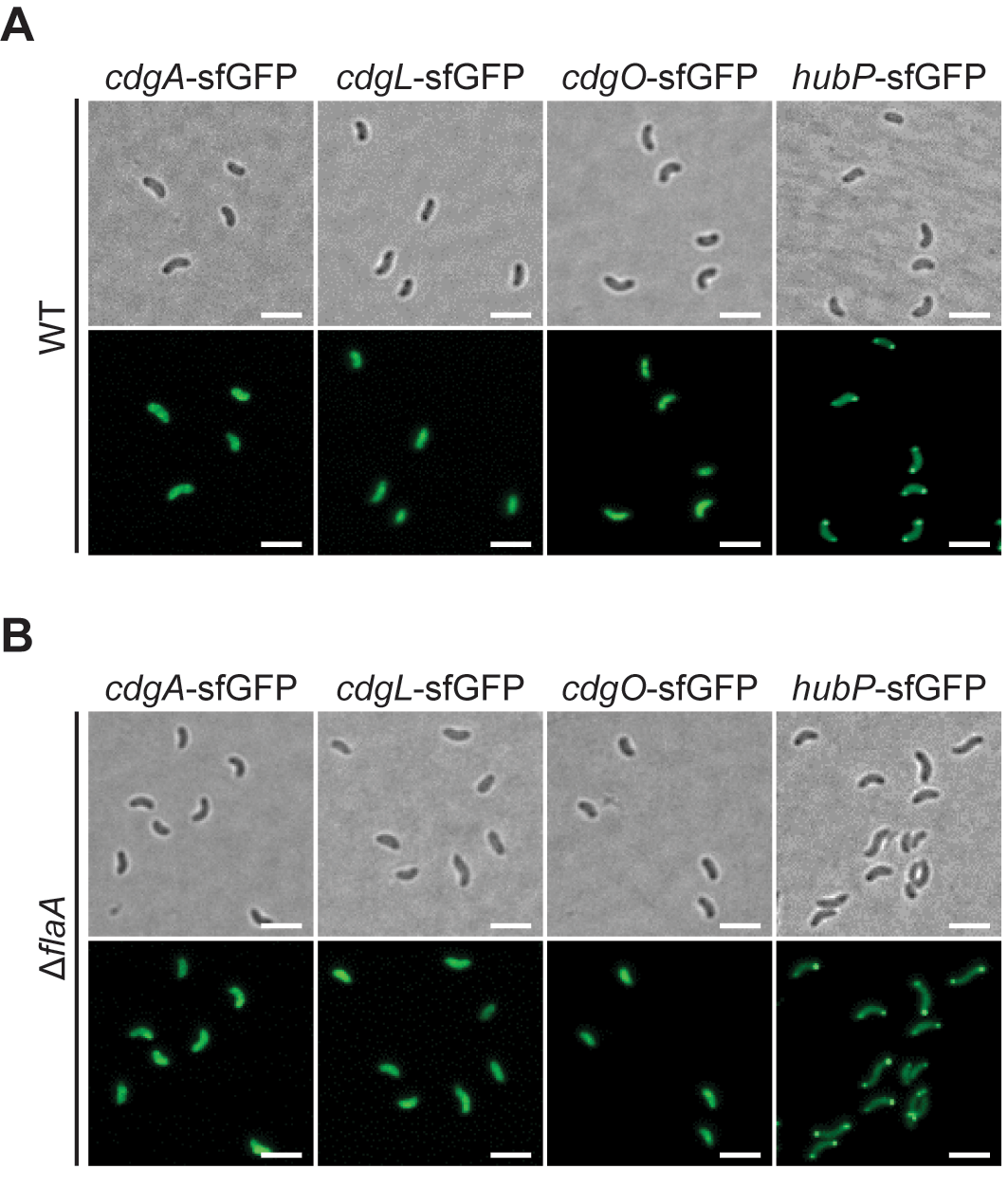

Supplement: S3 Fig — Representative bright-field and fluorescence microscopy images showing the intracellular distributions of superfolder GFP-labeled CdgA, CdgL, and CdgO in individual cells from the A) WT and B) ΔflaA genetic backgrounds. HubP was used as a positive control for polar localization. Scale bars = 5 μm. (TIF) [file pgen.1008703.s003.tif]

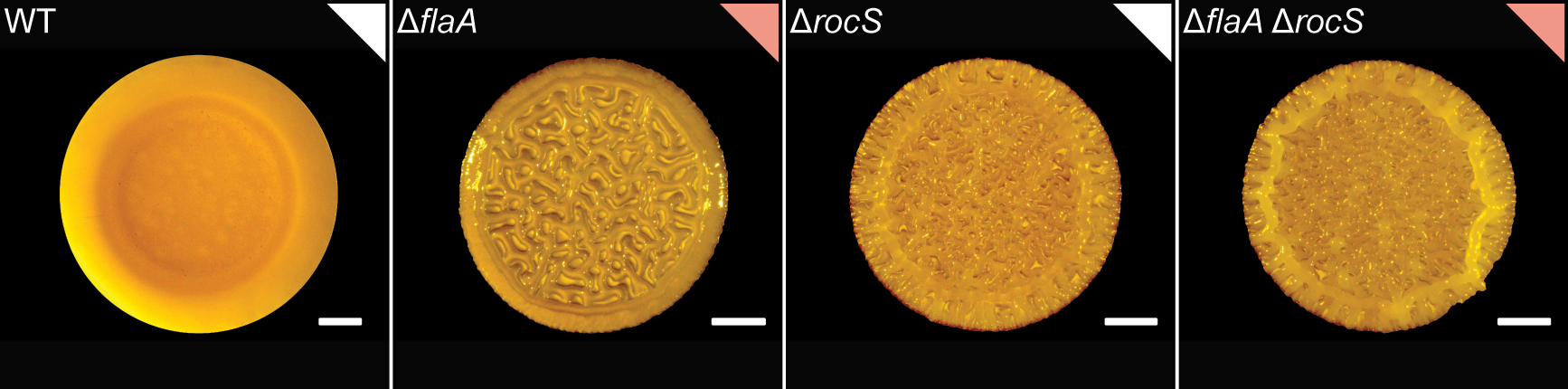

Supplement: S4 Fig — Representative images of the colony morphologies of the WT, ΔrocS, ΔflaA, and ΔflaA ΔrocS strains. (TIF) [file pgen.1008703.s004.tif]

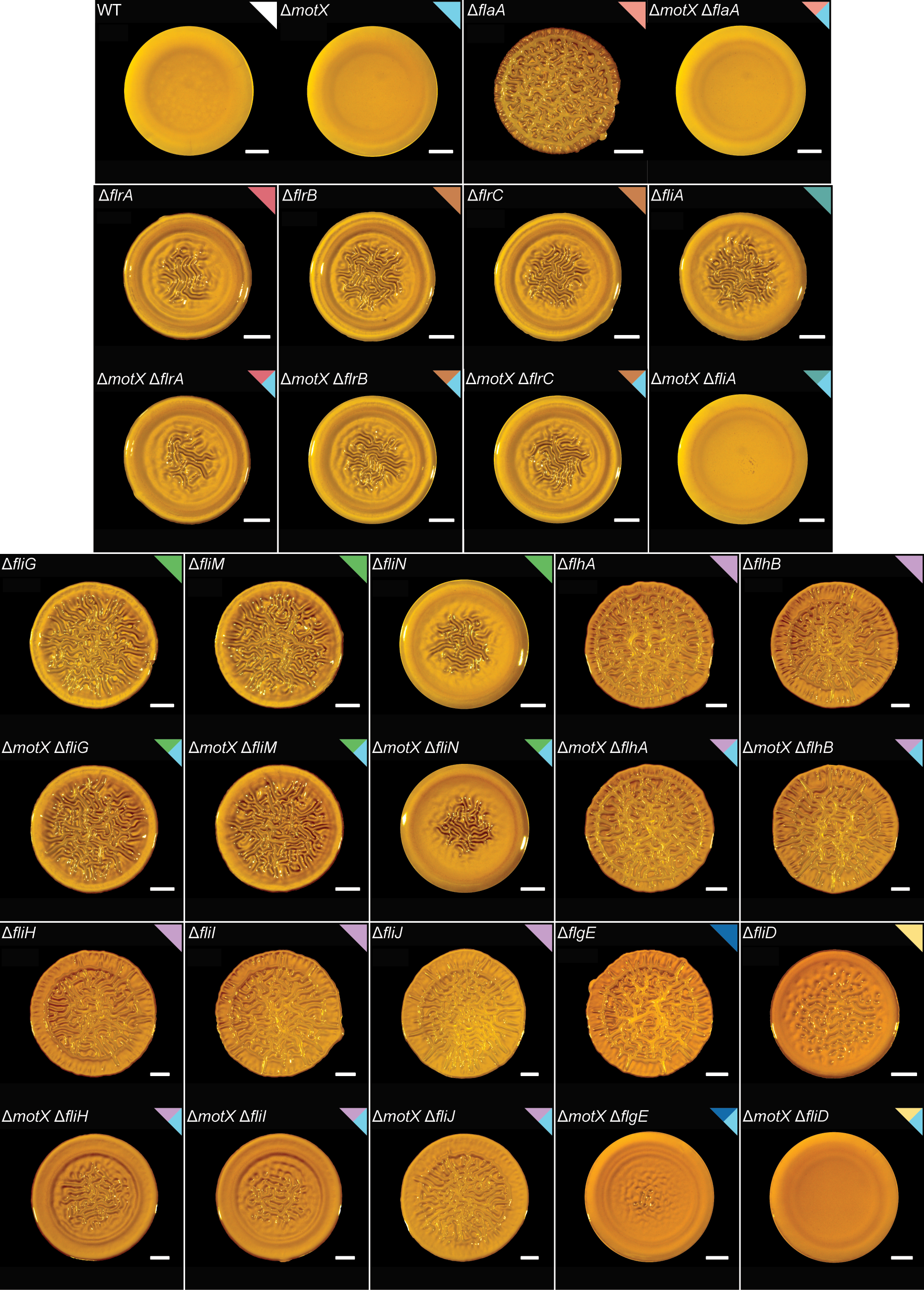

Supplement: S5 Fig — Representative images of the colony morphologies of the WT strain and a variety of flagellar mutants also lacking the T-ring gene motX (the same images of the single mutants are shown in Fig 7). (TIF) [file pgen.1008703.s005.tif]

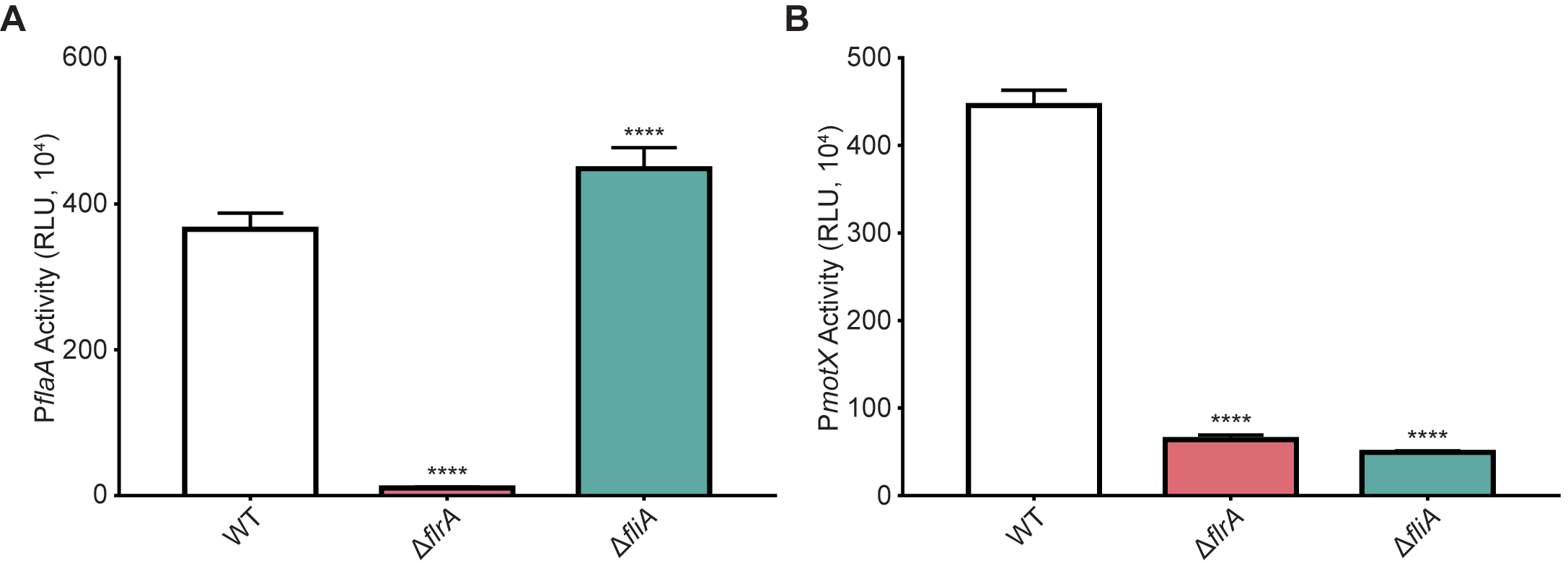

Supplement: S6 Fig — Bar graph of means and standard deviations of RLU obtained from the transcription of A) flaA-luxCDABE or B) motX-luxCDABE in exponentially grown cells. Means obtained from at least three independent biological replicates were compared to the WT strain with a one-way ANOVA and Dunnett’s multiple-comparison test. Adjusted P values ≤ 0.05 were deemed significant. *** p ≤ 0.001 **** p ≤ 0.0001. (TIF) [file pgen.1008703.s006.tif]
