# Supplementary material for: Reciprocal c-di-GMP signaling: Incomplete flagellum biogenesis triggers c-di-GMP signaling pathways that promote biofilm formation
Source: PLoS Genet. 2020 Mar 16;16(3):e1008703. doi: 10.1371/journal.pgen.1008703 (PMC7098655; doi:10.1371/journal.pgen.1008703)
Supplement: S1 Table — (PDF) [file pgen.1008703.s007.pdf]

S1 Table. COMSTAT2 quantification of Biofilm competition parameters.

Strain / Treatment	Biomass (μm3/μm2)	
	Competitor	WT
1 hour		
WT vs. WT	0.0154 (0.0050)	0.0125 (0.0036)
Δ*flaA* vs. WT	0.0207 (0.0018)	0.0155 (0.0008)
Δ*motX* vs. WT	0.0010 (0.0007)	0.0129 (0.0020)
Δ*flaA*Δ*motX* vs. WT	0.0009 (0.0008)	0.0119 (0.0039)
3 hour		
WT vs. WT	0.0701 (0.0206)	0.0589 (0.0166)
Δ*flaA* vs. WT	0.0768 (0.0196)	0.0826 (0.0116)
Δ*motX* vs. WT	0.0054 (0.0041)	0.0673 (0.0125)
Δ*flaA*Δ*motX* vs. WT	0.0048 (0.0039)	0.0706 (0.0255)
6 hour		
WT vs. WT	14.42 (1.68)	8.22 (2.81)
Δ*flaA* vs. WT	13.96 (1.20)	7.37 (6.93)
Δ*motX* vs. WT	0.99 (0.79)	11.10 (5.30)
Δ*flaA*Δ*motX* vs. WT	0.91 (0.67)	10.98 (4.18)
24 hour		
WT vs. WT	39.93 (6.31)	26.96 (2.65)
Δ*flaA* vs. WT	50.99 (9.16)	27.78 (9.62)
Δ*motX* vs. WT	3.01 (1.45)	41.54 (3.51)
Δ*flaA*Δ*motX* vs. WT	2.41 (1.10)	41.23 (3.09)
	Surface Area (μm2)	
	Competitor	WT
1 hour		
WT vs. WT	5.89E+03 (1.20E+03)	5.00E+03 (1.13E+03)
Δ*flaA* vs. WT	8.05E+03 (6.54E+02)	6.18E+03 (5.72E+02)
Δ*motX* vs. WT	4.82E+02 (3.64E+02)	4.55E+03 (6.36E+02)
Δ*flaA*Δ*motX* vs. WT	4.95E+02 (4.11E+02)	4.77E+03 (1.06E+03)
3 hour		
WT vs. WT	1.93E+04 (5.50E+03)	1.76E+04 (5.37E+03)
Δ*flaA* vs. WT	2.11E+04 (6.74E+03)	2.25E+04 (5.79E+03)
Δ*motX* vs. WT	1.67E+03 (1.33E+03)	1.79E+04 (4.14E+03)
Δ*flaA*Δ*motX* vs. WT	1.69E+03 (1.38E+03)	2.06E+04 (7.37E+03)

6 hour		
WT vs. WT	2.50E+06 (8.06E+05)	1.96E+06 (9.17E+05)
Δ*flaA* vs. WT	2.44E+06 (4.05E+05)	1.47E+06 (1.37E+06)
Δ*motX* vs. WT	2.25E+05 (1.68E+05)	2.48E+06 (1.68E+06)
Δ*flaA*Δ*motX* vs. WT	2.07E+05 (1.45E+05)	2.51E+06 (1.10E+06)
24 hour		
WT vs. WT	3.64E+06 (4.90E+05)	3.71E+06 (4.86E+05)
Δ*flaA* vs. WT	4.48E+06(1.12E+06)	4.82E+06 (1.21E+06)
Δ*motX* vs. WT	5.45E+05 (2.00E+05)	3.77E+06 (6.15E+04)
Δ*flaA*Δ*motX* vs. WT	6.10E+05 (2.24E+05)	4.41E+06 (7.02E+05)
	Micro-colonies at Substratum (Total number)	
	Competitor	WT
1 hour		
WT vs. WT	31	17
Δ*flaA* vs. WT	32	36
Δ*motX* vs. WT	0	17
Δ*flaA*Δ*motX* vs. WT	0	3
3 hour		
WT vs. WT	127	92
Δ*flaA* vs. WT	446	220
Δ*motX* vs. WT	1	69
Δ*flaA*Δ*motX* vs. WT	0	62
6 hour		
WT vs. WT	411	168
Δ*flaA* vs. WT	693	193
Δ*motX* vs. WT	57	126
Δ*flaA*Δ*motX* vs. WT	46	164
	Micro-colonies at Substratum (Average Volume (μm2))	
	Competitor	WT
1 hour		
WT vs. WT	186.14 (0.25)	216.89 (45.59)
Δ*flaA* vs. WT	120.04 (16.85)	211.85 (69.00)
Δ*motX* vs. WT	n/a	245.50 (23.02)
Δ*flaA*Δ*motX* vs. WT	n/a	205.13 (16.89)

3 hour		
WT vs. WT	159.52 (51.30)	209.39 (118.51)
Δ*flaA* vs. WT	128.75 (15.93)	188.96 (38.90)
Δ*motX* vs. WT	157.17 (n/a)	244.08 (159.04)
Δ*flaA*Δ*motX* vs. WT	n/a	162.73 (66.63)
6 hour		
WT vs. WT	468.45 (221.17)	513.63 (337.30
Δ*flaA* vs. WT	256.58 (51.41)	593.28 (264.75)
Δ*motX* vs. WT	116.30 (18.29)	677.76 (485.98)
Δ*flaA*Δ*motX* vs. WT	105.20 (11.39)	485.15 (355.43)
	Thickness-Biomass (Average (μm))	
	Competitor	WT
6 hour		
WT vs. WT	32.85 (4.40)	29.95 (3.79)
Δ*flaA* vs. WT	31.73 (3.51)	26.60 (1.56)
Δ*motX* vs. WT	27.72 (4.66)	26.32 (4.36)
Δ*flaA*Δ*motX* vs. WT	26.30 (2.73)	22.41 (2.36)
24 hour		
WT vs. WT	47.39 (7.76)	37.48 (3.05)
Δ*flaA* vs. WT	57.57 (10.62)	43.17 (12.38)
Δ*motX* vs. WT	27.93 (3.94)	43.25 (3.59)
Δ*flaA*Δ*motX* vs. WT	31.13 (4.24)	44.64 (4.59)
	Thickness-Biomass (Max (μm))	
	Competitor	WT
6 hour		
WT vs. WT	57.47 (13.92)	57.47 (13.92)
Δ*flaA* vs. WT	50.67 (4.42)	50.67 (4.42)
Δ*motX* vs. WT	40.90 (2.54)	42.92 (3.50)
Δ*flaA*Δ*motX* vs. WT	38.27 (2.46)	37.08 (2.73)
24 hour		
WT vs. WT	83.27 (11.11)	82.87 (10.44)
Δ*flaA* vs. WT	90.83 (14.47)	90.54 (14.02)
Δ*motX* vs. WT	60.16 (4.98)	79.28 (10.84)
Δ*flaA*Δ*motX* vs. WT	60.95 (9.49)	72.91 (7.27)

	Roughness Coefficient	
	Competitor	WT
6 hour		
WT vs. WT	0.75 (0.20)	1.05 (0.34)
Δ*flaA* vs. WT	0.80 (0.05)	1.18 (0.57)
Δ*motX* vs. WT	1.89 (0.08)	0.71 (0.65)
Δ*flaA*Δ*motX* vs. WT	1.91 (0.07)	0.58 (0.30)
24 hour		
WT vs. WT	0.37 (0.01)	0.41 (0.10)
Δ*flaA* vs. WT	0.20 (0.04)	0.41 (0.14)
Δ*motX* vs. WT	1.74 (0.09)	0.19 (0.06)
Δ*flaA*Δ*motX* vs. WT	1.76 (0.07)	0.14 (0.02)
