# Supplementary material for: Reciprocal c-di-GMP signaling: Incomplete flagellum biogenesis triggers c-di-GMP signaling pathways that promote biofilm formation
Source: PLoS Genet. 2020 Mar 16;16(3):e1008703. doi: 10.1371/journal.pgen.1008703 (PMC7098655; doi:10.1371/journal.pgen.1008703)
Supplement: S2 Table — (PDF) [file pgen.1008703.s008.pdf]

Table S2. Transposon insertions that suppress biofilm matrix repression in Δ*flaA* Δ*motX*

Gene interrupted	Number of insertions	Gene function
flrA (VC2137)<br>*flrC* (VC2135)<br>*flrB* (VC2136)	12<br>5<br>4	Regulators of the flagellar hierarchy
flhA (VC2069)<br>*flhB* (VC2120)<br>*fliI* (VC2130)<br>*fliO* (VC2124)<br>*fliP* (VC2123)<br>*fliR* (VC2121)	12<br>5<br>1<br>1<br>5<br>2	Flagellum-specific transport machinery
fliF (VC2133)<br>*fliG* (VC2132)<br>*fliM* (VC2126)	2<br>2<br>2	M and C ring
fliE (VC2134)<br>*flgF* (VC2196)	2<br>1	Rod
hubP (VC0998)	1	Polar localization
cdgG (VC0900)<br>*rocS* (VC0653)	1<br>1	c-di-GMP signaling
VCA0106	1	Hypothetical
VC1329	1	Opacity related protein (D-galactose or D-glucose ABC transporter, permease protein/<br>hypothetical
BAH11101.1	1	Probably in a mobile element (prophage)
O3Y_07040	1	Probably in a mobile element (prophage)
