# Supplementary material for: Reciprocal c-di-GMP signaling: Incomplete flagellum biogenesis triggers c-di-GMP signaling pathways that promote biofilm formation
Source: PLoS Genet. 2020 Mar 16;16(3):e1008703. doi: 10.1371/journal.pgen.1008703 (PMC7098655; doi:10.1371/journal.pgen.1008703)
Supplement: S3 Table — (PDF) [file pgen.1008703.s009.pdf]

Table S3. Bacterial strains and plasmids used in this study.

Strain or plasmid	Relevant Genotype	Source
E. coli strain		
SM10 (λ*pir*)	*Thi, thr, leu, tonA, lacY, supE, recA,* RP4-2-Tc::Mu l*pir* Km^r π^+	(1)
V. cholerae strains		
Fy_Vc_1915	C6706, St^r	Ronald K. Taylor
Fy_Vc_10247	C6706 Δ*flaA*, St^r	This work
Fy_Vc_11134	C6706 Δ*flrA*, St^r	This work
Fy_Vc_10616	C6706 Δ*flrB*, St^r	This work
Fy_Vc_11136	C6706 Δ*flrC*, St^r	This work
Fy_Vc_11132	C6706 Δ*fliA*, St^r	This work
Fy_Vc_10632	C6706 Δ*fliG*, St^r	This work
Fy_Vc_10250	C6706 Δ*fliM*, St^r	This work
Fy_Vc_10634	C6706 Δ*fliN*, St^r	This work
Fy_Vc_10254	C6706 Δ*flhA*, St^r	This work
Fy_Vc_11124	C6706 Δ*flhB*, St^r	This work
Fy_Vc_11126	C6706 Δ*fliH*, St^r	This work
Fy_Vc_11128	C6706 Δ*fliI*, St^r	This work
Fy_Vc_11130	C6706 Δ*fliJ*, St^r	This work
Fy_Vc_13823	C6706 Δ*flgE*, St^r	This work
Fy_Vc_11142	C6706 Δ*fliD*, St^r	This work
Fy_Vc_12821	C6706 Δ*pomA*, St^r	This work
Fy_Vc_12825	C6706 Δ*pomB*, St^r	This work
Fy_Vc_10256	C6706 Δ*motX*, St^r	This work
Fy_Vc_13771	C6706 Δ*motY*, St^r	This work
Fy_Vc_10247	C6706 Δ*flaA*, St^r	This work
Fy_Vc_12827	C6706 Δ*flaA* Δ*pomA*, St^r	This work

Fy_Vc_12831	C6706 Δ*flaA* Δ*pomB*, St^r	This work
Fy_Vc_10249	C6706 Δ*flaA* Δ*motX*, St^r	This work
Fy_Vc_13773	C6706 Δ*flaA* Δ*motY*, St^r	This work
Fy_Vc_13799	C6706::*pomB*-D23E, St^r	This work
Fy_Vc_13801	C6706::*pomB*-D23N, St^r	This work
Fy_Vc_13803	C6706 Δ*flaA*::*pomB*-D23E, St^r	This work
Fy_Vc_13805	C6706 Δ*flaA*::*pomB*-D23N, St^r	This work
Fy_Vc_14289	C6706 Δ*nqrB*, St^r	This work
Fy_Vc_14291	C6706 Δ*nqrC*, St^r	This work
Fy_Vc_13479	C6706 Δ*flaA* Δ*nqrB*, St^r	This work
Fy_Vc_13481	C6706 Δ*flaA* Δ*nqrC*, St^r	This work
Fy_Vc_14287	C6706 Δ*sssA*, St^r	This work
Fy_Vc_13785	C6706 Δ*flaA* Δ*sssA*, St^r	This work
Fy_Vc_15289	C6706 Δ*mshA*, St^r	Yildiz lab collection
Fy_Vc_11157	C6706 Δ*flrA* Δ*motX*, St^r	This work
Fy_Vc_13809	C6706 Δ*flrB* Δ*motX*, St^r	This work
Fy_Vc_11159	C6706 Δ*flrC* Δ*motX*, St^r	This work
Fy_Vc_13807	C6706 Δ*fliA* Δ*motX*, St^r	This work
Fy_Vc_11153	C6706 Δ*fliG* Δ*motX*, St^r	This work
Fy_Vc_10261	C6706 Δ*fliM* Δ*motX*, St^r	This work
Fy_Vc_11155	C6706 Δ*fliN* Δ*motX*, St^r	This work
Fy_Vc_10638	C6706 Δ*flhA* Δ*motX*, St^r	This work
Fy_Vc_11168	C6706 Δ*flhB* Δ*motX*, St^r	This work
Fy_Vc_11170	C6706 Δ*fliH* Δ*motX*, St^r	This work
Fy_Vc_11172	C6706 Δ*fliI* Δ*motX*, St^r	This work
Fy_Vc_11174	C6706 Δ*fliJ* Δ*motX*, St^r	This work
Fy_Vc_13827	C6706 Δ*flgE* Δ*motX*, St^r	This work
FY_Vc_11190	C6706 Δ*fliD* Δ*motX*, St^r	This work
FY_Vc_10252	C6706 Δ*rocS*, St^r	This work
FY_Vc_13457	C6706 Δ*flaA* Δ*rocS*, St^r	This work

Fy_Vc_12833	C6706 Δ*flaA* Δ*VC1029* (*cdgB*) , St^{r}	This work
Fy_Vc_11151	C6706 Δ*flaA* Δ*VC1067* (*cdgH*) , St^{r}	This work
Fy_Vc_11149	C6706 Δ*flaA* Δ*VC1104* (*cdgK*) , St^{r}	This work
Fy_Vc_12835	C6706 Δ*flaA* Δ*VC1185*, St^{r}	This work
Fy_Vc_12837	C6706 Δ*flaA* Δ*VC1216*, St^{r}	This work
Fy_Vc_12840	C6706 Δ*flaA* Δ*VC1353*, St^{r}	This work
Fy_Vc_12843	C6706 Δ*flaA* Δ*VC1370*, St^{r}	This work
Fy_Vc_12845	C6706 Δ*flaA* Δ*VC1372*, St^{r}	This work
Fy_Vc_10659	C6706 Δ*flaA* Δ*VC1376* (*cdgM*) , St^{r}	This work
Fy_Vc_12847	C6706 Δ*flaA* Δ*VC1593*, St^{r}	This work
Fy_Vc_13487	C6706 Δ*flaA* Δ*VC1599*, St^{r}	This work
Fy_Vc_12849	C6706 Δ*flaA* Δ*VC2224*, St^{r}	This work
Fy_Vc_10657	C6706 Δ*flaA* Δ*VC2285* (*cdgL*) , St^{r}	This work
Fy_Vc_12851	C6706 Δ*flaA* Δ*VC2370*, St^{r}	This work
Fy_Vc_12853	C6706 Δ*flaA* Δ*VC2454* (*vpvC*) , St^{r}	This work
Fy_Vc_12855	C6706 Δ*flaA* Δ*VC2697*, St^{r}	This work
Fy_Vc_12209	C6706 Δ*flaA* Δ*VCA0050-49* (*cdgO*) , St^{r}	This work
Fy_Vc_10655	C6706 Δ*flaA* Δ*VCA0074* (*cdgA*) , St^{r}	This work
Fy_Vc_12857	C6706 Δ*flaA* Δ*VCA0165*, St^{r}	This work
Fy_Vc_12859	C6706 Δ*flaA* Δ*VCA0217*, St^{r}	This work
Fy_Vc_12861	C6706 Δ*flaA* Δ*VCA0557*, St^{r}	This work
Fy_Vc_12863	C6706 Δ*flaA* Δ*VCA0560*, St^{r}	This work
Fy_Vc_12865	C6706 Δ*flaA* Δ*VCA0697* (*cdgD*) , St^{r}	This work
Fy_Vc_12867	C6706 Δ*flaA* Δ*VCA0848*, St^{r}	This work
Fy_Vc_12869	C6706 Δ*flaA* Δ*VCA0939* (*cdgP*) , St^{r}	This work
Fy_Vc_13119	C6706 Δ*flaA* Δ*VCA0956* (*cdgF*) , St^{r}	This work
Fy_Vc_12871	C6706 Δ*flaA* Δ*VCA0960*, St^{r}	This work
Fy_Vc_13115	C6706 Δ*flaA* Δ*VCA0965*, St^{r}	This work
Fy_Vc_10628	C6706 Δ*cdgA*, St^{r}	This work
Fy_Vc_10624	C6706 Δ*cdgL*, St^{r}	This work

Fy_Vc_12209	C6706 Δ*cdgO*, St^r	This work
Fy_Vc_14259	C6706 Δ*cdgLO*, St^r	This work
Fy_Vc_13467	C6706 Δ*cdgALO*, St^r	This work
Fy_Vc_13751	C6706 Δ*flaA* Δ*cdgALO*, St^r	This work
Fy_Vc_14261	C6706 Δ*flaA* Δ*cdgLO*	This work
Fy_Vc_14216	C6706 Δ*flrA* Δ*cdgALO*, St^r	This work
Fy_Vc_14218	C6706 Δ*flrC* Δ*cdgALO*, St^r	This work
Fy_Vc_14220	C6706 Δ*fliA* Δ*cdgALO*, St^r	This work
Fy_Vc_14222	C6706 Δ*fliG* Δ*cdgALO*, St^r	This work
Fy_Vc_14224	C6706 Δ*flhA* Δ*cdgALO*, St^r	This work
Fy_Vc_14226	C6706 Δ*fliH* Δ*cdgALO*, St^r	This work
Fy_Vc_14043	C6706::*hubP*-sfGFP, St^r	This work
Fy_Vc_14047	C6706::*cdgA*-sfGFP, St^r	This work
Fy_Vc_14071	C6706::*cdgL*-sfGFP, St^r	This work
Fy_Vc_14049	C6706::*cdgO*-sfGFP, St^r	This work
Fy_Vc_10640	C6706 Δ*flaA* Δ*vpsR*, St^r	
Fy_Vc_15545	C6706 Δ*flaA* Δ*vpsR*::*vpsR*-D59E, St^r	
Fy_Vc_15645	C6706 Δ*flaA* Δ*vpsR*::*vpsR*-D59A, St^r	
Fy_Vc_14039	C6706 Δ*flaA* Δ*motX* Δ*vpsR*, St^r	This work
Fy_Vc_14067	C6706 Δ*flaA* Δ*motX* Δ*vpsR*::*vpsR*-D59A, St^r	This work
Fy_Vc_14069	C6706 Δ*flaA* Δ*motX* Δ*vpsR*::*vpsR*-D59E, St^r	This work
Fy_Vc_13829	C6706 Δ*flgM*, St^r	This work
Fy_Vc_11138	C6706 Δ*hapR*, St^r	This work
Fy_Vc_12697	C6706 Δ*flaA* Δ*hapR*	This work
Fy_Vc_13453	C6706 Δ*flaA* Δ*motX* Δ*hapR*	This work
Plasmids		

pFY_23 (pGP704sac28)	pGP704 derivative; mob-oriT, sacB, Ap^r	G. Schoolnik
pFY_4394	pGP704sac28-*flaA*, Ap^r	This work
pFY_4393	pGP704sac28-*flrA*, Ap^r	This work
pFY_4298	pGP704sac28-*flrB*, Ap^r	This work
pFY_4392	pGP704sac28-*flrC*, Ap^r	This work
pFY_4391	pGP704sac28-*fliA*, Ap^r	This work
pFY_4288	pGP704sac28-*fliG*, Ap^r	This work
pFY_4396	pGP704sac28-*fliM*, Ap^r	This work
pFY_4287	pGP704sac28-*fliN*, Ap^r	This work
pFY_4305	pGP704sac28-*flhA*, Ap^r	This work
pFY_4397	pGP704sac28-*flhB*, Ap^r	This work
pFY_4399	pGP704sac28-*fliH*, Ap^r	This work
pFY_4401	pGP704sac28-*fliI*, Ap^r	This work
pFY_4403	pGP704sac28-*fliJ*, Ap^r	This work
pFY_5915	pGP704sac28-*flgE*, Ap^r	This work
pFY_4408	pGP704sac28-*fliD*, Ap^r	This work
pFY_5309	pGP704sac28-*pomA*, Ap^r	This work
pFY_5310	pGP704sac28-*pomB*, Ap^r	This work
pFY_5883	pGP704sac28-*motX*, Ap^r	This work
pFY_5550	pGP704sac28-*motY*, Ap^r	This work
pFY_5778	pGP704sac28-*pomB*-D23E, Ap^r	This work
pFY_5779	pGP704sac28-*pomB*-D23N, Ap^r	This work
pFY_5574	pGP704sac28-VC2294 (*nqrB*), Ap^r	This work
pFY_5573	pGP704sac28-VC2293 (*nqrC*), Ap^r	This work
pFY_5869	pGP704sac28-*sssA*	This work
pFY_5868	pGP704sac28-VC0303	This work
pFY_4405	pGP704sac28-*rocS*, Ap^r	(2)

pFY_4805	pGP704sac28-VC1029 (*cdgB*), Ap^r	(2)
pFY_4300	pGP704sac28-*cdgH*, Ap^r	(3)
pFY_4301	pGP704sac28-*cdgK*, Ap^r	(3,4)
pFY_4804	pGP704sac28-VC1185, Ap^r	(3,4)
pFY_4802	pGP704sac28-VC1216, Ap^r	(3,4)
pFY_4810	pGP704sac28-VC1353, Ap^r	(3,4)
pFY_4806	pGP704sac28-VC1370, Ap^r	(3,4)
pFY_4803	pGP704sac28-VC1372, Ap^r	(3,4)
pFY_4303	pGP704sac28-*cdgM*	(3,4)
pFY_4811	pGP704sac28-VC1593, Ap^r	(3,4)
pFY_4812	pGP704sac28-VC1599, Ap^r	(3,4)
pFY_4813	pGP704sac28-VC2224, Ap^r	(3,4)
pFY_4302	pGP704sac28-*cdgL*, Ap^r	(3,4)
pFY_4814	pGP704sac28-VC2370, , Ap^r	(3,4)
pFY_4807	pGP704sac28-VC2454, , Ap^r	(3,4)
pFY_4815	pGP704sac28-VC2697, Ap^r	(3,4)
pFY_4734	pGP704sac28-VCA0050-49, Ap^r	This work
pFY_4299	pGP704sac28-*cdgA*, Ap^r	(2)
pFY_4817	pGP704sac28-VCA0165, Ap^r	(3,4)
pFY_4808	pGP704sac28-VCA0217, Ap^r	(3,4)
pFY_4818	pGP704sac28-VCA0557, Ap^r	(3,4)
pFY_4819	pGP704sac28-VCA0560, Ap^r	(3,4)
pFY_4820	pGP704sac28-VCA0697 (*cdgD*), Ap^r	(2)
pFY_4821	pGP704sac28-VCA0848, Ap^r	(3,4)
pFY_4822	pGP704sac28-VCA0939, Ap^r	(3,4)
pFY_4823	pGP704sac28-VCA0956, Ap^r	(3,4)
pFY_4809	pGP704sac28-VCA0960, Ap^r	(3,4)
pFY_5546	pGP704sac28-VCA0965, Ap^r	(3,4)
pFY_5676	pGP704sac28-*vipA-sfGFP*, Ap^r	Yildiz lab collection
pFY_5790	pGP704sac28-*hubP-sfGFP*, Ap^r	This work

pFY_5792	pGP704sac28-*cdgA*-*sfGFP*, Ap^r	This work
pFY_5799	pGP704sac28-*cdgL*-*sfGFP*, Ap^r	This work
pFY_5793	pGP704sac28-*cdgO*-*sfGFP*, Ap^r	This work
pFY_4395	pGP704sac28-*hapR*, Ap^r	(3)
pFY_4390	pGP704sac28-*vpsR*, Ap^r	(5)
pFY_5797	pGP704sac28-*vpsR*-D59A, Ap^r	This work
pFY_5798	pGP704sac28-*vpsR*-D59E, Ap^r	This work
pFY_5752	Plasmid expressing GST-*fliI*	This work
pFY_691 (pBBRlux)	*luxCDABE*-based promoter fusion vector; Cm^r	(6)
pFY_4290	pBBRlux-P*vpsL*; Cm^r	This work
pFY_1122	pBBRlux-P*flaA*; Cm^r	(7)
pFY_6409	pBBRlux-P*motX*; Cm^r	This work
pFY_1303 (pMMB67EH)	Expression vector harboring *lacI* and containing a Ptac promoter, Ap^r.	Yildiz lab collection
pFY_4289	C-di-GMP biosensor cloned in pMMB67EH, Ap^r.	This work
pFY_4535	C-di-GMP biosensor containing the *hok*/*sok* region from pXB300, Gm^r	(7)

References

1. Simon R, Priefer U, Pühler A. A Broad Host Range Mobilization System for In Vivo Genetic Engineering: Transposon Mutagenesis in Gram Negative Bacteria. Bio/Technology [Internet]. 1983 Nov 1 [cited 2017 Jul 5];1(9):784–91. Available from: http://www.nature.com/doifinder/10.1038/nbt1183-784
2. Lim B, Beyhan S, Meir J, Yildiz FH. Cyclic-diGMP signal transduction systems in Vibrio cholerae: modulation of rugosity and biofilm formation. Mol Microbiol [Internet]. 2006 Apr [cited 2016 Sep 25];60(2):331–48. Available from: http://www.ncbi.nlm.nih.gov/pubmed/16573684
3. Beyhan S, Odell LS, Yildiz FH. Identification and characterization of cyclic diguanylate signaling systems controlling rugosity in Vibrio cholerae. J Bacteriol [Internet]. 2008 Nov [cited 2016 Sep 25];190(22):7392–405. Available from: http://www.ncbi.nlm.nih.gov/pubmed/18790873
4. Liu X, Beyhan S, Lim B, Linington RG, Yildiz FH. Identification and characterization of a phosphodiesterase that inversely regulates motility and biofilm formation in Vibrio cholerae. J Bacteriol [Internet]. 2010 Sep [cited 2016 Sep 25];192(18):4541–52. Available from: http://www.ncbi.nlm.nih.gov/pubmed/20622061
5. Yildiz FH, Dolganov NA, Schoolnik GK. VpsR , a Member of the Response Regulators of the Two-Component Regulatory Systems , Is Required for Expression of vps Biosynthesis Genes and EPS ETr -Associated Phenotypes in Vibrio cholerae O1 El Tor. J Bacteriol. 2001 Mar;183(5):1716–26.
6. Lenz DH, Mok KC, Lilley BN, Kulkarni R V, Wingreen NS, Bassler BL. The Small RNA Chaperone Hfq and Multiple Small RNAs Control Quorum Sensing in Vibrio harveyi and Vibrio cholerae. Cell [Internet]. 2004 Jul 9 [cited 2017 Mar 2];118(1):69–82. Available from: http://www.ncbi.nlm.nih.gov/pubmed/15242645
7. Zamorano-Sánchez D, Xian W, Lee CK, Salinas M, Thongsomboon W, Cegelski L, et al. Functional Specialization in *Vibrio cholerae* Diguanylate Cyclases: Distinct Modes of Motility Suppression and c-di-GMP Production. Peter Greenberg E, editor. MBio [Internet]. 2019 Apr 23 [cited 2019 Jul 7];10(2). Available from: http://www.ncbi.nlm.nih.gov/pubmed/31015332
