# Supplementary material for: Reciprocal c-di-GMP signaling: Incomplete flagellum biogenesis triggers c-di-GMP signaling pathways that promote biofilm formation
Source: PLoS Genet. 2020 Mar 16;16(3):e1008703. doi: 10.1371/journal.pgen.1008703 (PMC7098655; doi:10.1371/journal.pgen.1008703)
Supplement: S1 Text — (PDF) [file pgen.1008703.s010.pdf]

Text S1. Extended Methods

Analysis of protein localization. Analysis of protein localization. Overnight cultures of strains containing the chromosomally tagged sfGFP were diluted 1:50 in DASW (Defined Artificial Sea Water; 234 mM NaCl, 27.5 mM $MgSO_4$, 1.5 mM $NaHCO_3$, 4.95 mM $CaCl_2$, 5.15 mM KCl, 0.07 mM $Na_2B_4O_7$, 0.05 mM SrCl, 0.015 mM NaBr, 0.001 mM NaI, 0.013 mM LiCl, 18.7 mM NH_4Cl, 0.187 mM K_2HPO_4, 50 mM HEPES, pH 7.4) and 4 µL of the diluted culture were pipetted on 40 µL agarose pads (1% agarose in DASW) on glass slides. Cover slips were placed over the agarose pad and sealed utilizing Valap (1:1:1 [w/w/w/] mixture of Lanoline, Paraffin, and Vaseline). The bacterial cells were imaged utilizing the Zeiss Zen Blue.
